# Transmission of SARS-CoV-2 Associated with Cruise Ship Travel: A Systematic Review

**DOI:** 10.3390/tropicalmed7100290

**Published:** 2022-10-09

**Authors:** Elena Cecilia Rosca, Carl Heneghan, Elizabeth A. Spencer, Jon Brassey, Annette Plüddemann, Igho J. Onakpoya, David Evans, John M. Conly, Tom Jefferson

**Affiliations:** 1 Department of Neurology, Victor Babes University of Medicine and Pharmacy, Piata Eftimie Murgu 2, 300041 Timisoara, Romania; 2 Centre for Evidence Based Medicine, Nuffield Department of Primary Care Health Sciences, University of Oxford, Radcliffe Observatory Quarter, Oxford OX2 6GG, UK; 3 Trip Database Ltd., Glasllwch Lane, Newport NP20 3PS, UK; 4 Department for Continuing Education, University of Oxford, Rewley House, 1 Wellington Square, Oxford OX1 2JA, UK; 5 Li Ka Shing Institute of Virology, and Department of Medical Microbiology & Immunology, University of Alberta, Edmonton, AB T6G 2R3, Canada; 6 Departments of Medicine, Microbiology, Immunology & Infectious Diseases, and Pathology & Laboratory Medicine, Synder Institute for Chronic Diseases and O’Brien Institute for Public Health, Cumming School of Medicine, University of Calgary and Alberta Health Services, Calgary, AB T2N 4N1, Canada

**Keywords:** COVID-19, SARS-CoV-2, transmission, cruise ship, environment, systematic review

## Abstract

Background: Maritime and river travel may be associated with respiratory viral spread via infected passengers and/or crew and potentially through other transmission routes. The transmission models of SARS-CoV-2 associated with cruise ship travel are based on transmission dynamics of other respiratory viruses. We aimed to provide a summary and evaluation of relevant data on SARS-CoV-2 transmission aboard cruise ships, report policy implications, and highlight research gaps. Methods: We searched four electronic databases (up to 26 May 2022) and included studies on SARS-CoV-2 transmission aboard cruise ships. The quality of the studies was assessed based on five criteria, and relevant findings were reported. Results: We included 23 papers on onboard SARS-CoV-2 transmission (with 15 reports on different aspects of the outbreak on Diamond Princess and nine reports on other international cruises), 2 environmental studies, and 1 systematic review. Three articles presented data on both international cruises and the Diamond Princess. The quality of evidence from most studies was low to very low. Index case definitions were heterogeneous. The proportion of traced contacts ranged from 0.19 to 100%. Studies that followed up >80% of passengers and crew reported attack rates (AR) up to 59%. The presence of a distinct dose–response relationship was demonstrated by findings of increased ARs in multi-person cabins. Two studies performed viral cultures with eight positive results. Genomic sequencing and phylogenetic analyses were performed in individuals from three cruises. Two environmental studies reported PCR-positive samples (cycle threshold range 26.21–39.00). In one study, no infectious virus was isolated from any of the 76 environmental samples. Conclusion: Our review suggests that crowding and multiple persons per cabin were associated with an increased risk of transmission on cruise ships. Variations in design, methodology, and case ascertainment limit comparisons across studies and quantification of transmission risk. Standardized guidelines for conducting and reporting studies on cruise ships of acute respiratory infection transmission should be developed.

## 1. Introduction

The emergence of the severe acute respiratory syndrome coronavirus 2 (SARS-CoV-2) was declared a public health emergency by the World Health Organization (WHO) in January 2020 and declared a global pandemic in March 2020. The SARS-CoV-2 spread rapidly, and national and international agencies, including the WHO, worked to develop prevention, control, and management measures on several fronts, aiming to control the pandemic by suppressing the transmission of the virus and preventing associated illness and death [1,2]. Nevertheless, the transmission routes of SARS-CoV-2 are not entirely understood and the public health measures for limiting transmission are based on the best available information.

Maritime and river travel, including cruise ships, has been associated with many viral infections that can spread to passengers and crew. Cruise ships may facilitate viral transmission within the relatively confined environments on ships and with passengers and crew being in close proximity to one another for extended periods [3,4]. Depending on the type of virus, the onboard transmission and consequent development of outbreaks may be facilitated by direct person-to-person contact, contact with contaminated surfaces, and through water and foodborne routes.

Shipboard activities generally involve gathering large numbers of people together, including for dining, games, movies, tours, concerts, gambling, parties, and dancing; these settings increase the chance of contact between passengers and crew [5]. Also, cruise travel includes frequent layovers at ports of call where new crew and travelers can board, allowing viral transmission from infected individuals to susceptible persons [3]. Furthermore, modern cruise ships accommodate numerous travelers, often including older passengers with medical comorbidities. The incubation period and subsequent period of maximum infectivity of several infectious diseases fall within the average cruise duration of 6 days [6].

During February–March 2020, SARS-CoV-2 outbreaks occurred during several well-publicized cruise ship voyages, reporting more than 800 cases among passengers and crew [7]. Consequently, in February 2020, several national and international organizations recommended the deferral of ship travel worldwide and guidance on managing SARS-CoV-2 cases and outbreaks aboard ships [8,9]. In March 2020, Cruise Lines International Association announced a 30-day voluntary suspension of cruise operations in the United States (USA). Also, CDC issued a No Sail Order for cruise ships, suspending operation in US waters, which was renewed on 9 April, effective 15 April 2020 [10]. One year later, guidance was provided on the gradual and safe resumption of cruise ship operations [11,12].

Given the ongoing need to assess the circumstances and modes of transmission of SARS-CoV-2, early cruise ship travel transmission models were based on knowledge of the transmission dynamics of other respiratory viral infections, particularly influenza. Consequently, there is a need to continuously and systematically review publicly available studies to enhance our understanding of the modes of transmission and consequent preventive measures on cruise ships.

### Objectives

We aimed to provide an evidence-informed summary and evaluation of relevant data regarding SARS-CoV-2 transmission aboard cruise ships, discuss policy implications, and highlight research gaps. We set out to address the following questions:

1. Is SARS-CoV-2 transmitted aboard cruise ships?
2. If so, what is/are the predominant mode(s) of transmission?
3. Are there particular circumstances that facilitate transmission (practices, ship layout, or populations)?

## 2. Materials and Methods

The present work is an open evidence review on SARS-CoV-2 transmission associated with cruise ship travel. We developed the present protocol [13] based on a previous protocol used for a series of systematic reviews on the transmission dynamics of COVID-19 (Supplementary Materials File S1).

We searched four electronic databases: LitCovid, medRxiv, Google Scholar, and WHO COVID-19 database, up to 26 May 2022. Search terms were COVID-19, SARS-CoV-2, transmission, ship, and appropriate synonyms (Supplementary Materials File S2). We also searched for additional studies through checking reference lists of relevant articles, including reviews. We did not set any language restrictions.

We included studies reporting on SARS-CoV-2 transmission aboard cruise ships, from passengers and crew to passengers or crew. We considered any potential modes of transmission, including long-range airborne, close contact, droplet, fomite, fecal-oral, and/or mixed routes. We included studies of any design except predictive or modeling studies. If two or more papers presented the same data, we included only the most comprehensive report. Articles were excluded if they did not report primary data.

One reviewer (ECR) extracted data from included studies, and these were independently checked by a second reviewer (EAS). Disagreements were resolved via consensus.

The quality of included human and environmental studies was assessed based on a modified QUADAS-2 tool using five criteria. A detailed presentation of the quality assessment is presented in the review protocol [13].

As the included studies were not primarily designed as diagnostic accuracy studies, we adapted the QUADAS-2 tool. Two reviewers (ECR, EAS) independently assessed the quality of included studies. We resolved disagreements via consensus. We did not formally assess the quality of included systematic reviews but summarized their findings.

For studies generating a hypothesis of onboard SARS-CoV-2 transmission, we also assessed the strength of evidence of each study depending on the methods used to investigate the virus transmission [14]. The results are presented in tabular format. Where relevant, we reported results of specific subgroups. Meta-analysis was only considered appropriate if minimal heterogeneity was found among included studies.

## 3. Results

Our searches identified 658 studies, of which we assessed the full text of 79 studies (Supplementary Materials File S3). We excluded 53 studies (see Supplementary Materials File S3 and File S4 for the reasons for exclusion). In total, we included 26 articles: 2 environmental studies [15,16], 23 papers considering transmission of SARS-CoV-2 aboard cruise ships [6,7,17,18,19,20,21,22,23,24,25,26,27,28,29,30,31,32,33,34,35,36,37], and one systematic review [38] (Supplementary Materials File S5). The main characteristics of the included studies are presented in Table 1 and Table 2.

Among 23 papers reporting on onboard transmission of SARS-CoV-2, 15 articles presented different aspects of the outbreak on the Diamond Princess (DP) cruise ship [7,17,19,20,21,23,24,25,26,27,29,30,31,35,37]. Nine articles reported on other international ships [6,7,18,22,28,32,33,34,36]. However, three of the latter papers also included the DP [6,7,18].

Six studies reporting on the DP outbreak presented data focusing on repatriated citizens from the US [7,26,29], Hong Kong [21], Israel [17], and Australia [35]. Although there were similarities between results of the studies on the US repatriated citizens [7,26,29], there were also some notable discrepancies, including the number of repatriated citizens, the number of SARS-CoV-2 cases, and importantly, the number of symptomatic and asymptomatic cases (Supplementary Materials File S6).

Our search identified one systematic review on SARS-CoV-2 outbreaks on ships [38]. The authors searched four databases up to 31 July 2020. They included 37 studies: 33 reported several aspects of SARS-CoV-2 outbreaks from cruise ships, three reported outbreaks of SARS-CoV-2 on navy vessels, and one study presented an outbreak on a cargo ship [38]. The review did not assess the risk of bias in the included studies.

### 3.1. Quality of Included Studies

None of the studies reported a published protocol. The risk of bias assessment of the included studies is presented in Table 3 and Table 4.

For the environmental studies [15,16], we found an adequate description of their methods, with sufficient detail to replicate the findings. Sample sources were clearly reported, and the analysis and reporting were considered appropriate; there were no concerns about their applicability. However, we found that neither study adequately addressed potential biases (Table 4).

Among studies presenting the DP outbreak, 6/15 (40%) presented a clearly defined setting [23,25,26,30,31,35]; 2/15 (13%) adequately described demographic characteristics and sampling procedures [21,24]. In 2/15 (13%) reports, strategy and duration of follow-up were sufficient for outcome assessments [25,31]. Transmission outcomes were adequately assessed for 2/15 (13%) studies [21,24]; data validity concerns were taken into consideration for 3/15 (20%) reports [24,25,31] (Table 4).

**Figure 1:**
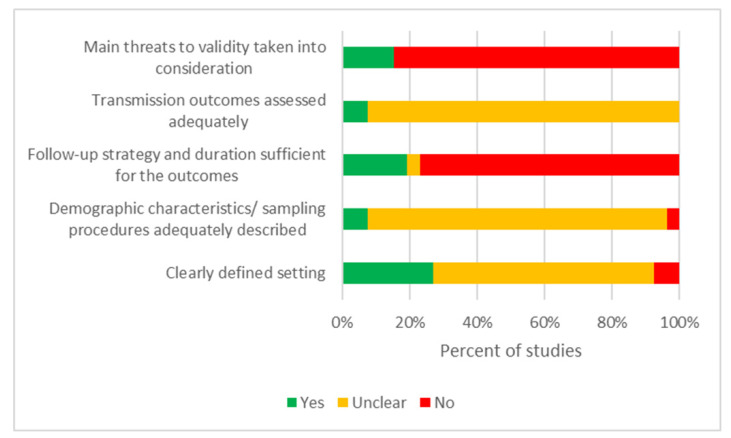
Risk of bias graph in studies on onboard transmission of SARS-CoV-2.

In studies reporting outbreaks on international ships, 1/11 (9%) reports clearly described the setting [34]. Demographic characteristics and sampling procedures were unclear in all studies. A comprehensive follow-up strategy was presented only in 3/11 (27%) studies [22,32,36]. Transmission outcomes were not adequately assessed by any authors (0/11), and only 1/11 (9%) studies took into consideration the validity of the data [22] (Table 4). The overall reporting quality across studies was considered low (Figure 1).

### 3.2. Environmental Studies

One study collected wastewater samples from a docked cruise ship [15] over a month after passengers disembarked, with only crew on board. Unconfirmed reports suggested that 24 infected individuals may have been on board in the days before sample collection. The wastewater samples were positive for SARS-CoV-2 RNA, but concentrations were near the assay limit of detection (Cycle quantification [Cq] 36.0–38.7). Greater concentrations were observed in the influent from the cruise ship than the effluent (Table 2).

The second study [16] collected environmental samples after the DP passengers and crew evacuated the cabins. SARS-CoV-2 RNA was detected in 58/601 samples (10%) from cabins with confirmed COVID-19 cases, 1–17 days after vacating the cabins. The authors found no difference in detection proportion between cabins of symptomatic (15%, 28/189; Cq 29.79–38.86) and asymptomatic persons (21%, 28/131; Cq 26.21–38.99). SARS-CoV-2 RNA was not detected in any cabin with no confirmed cases. SARS-CoV-2 RNA was not present in any of the air samples, or in common areas, except for one sample from an air hood in a corridor [16]. SARS-CoV-2 RNA was most often detected in bathrooms, on the floor around the toilets (39%, 13/33; Cq 26.21–37.62) and on bed pillows (34%, 11/32; Cq 34.61–38.99). No infectious virus was isolated from the 76 samples with SARS-CoV-2 RNA detected by RT-PCR [16].

### 3.3. Studies on the Onboard Transmission of SARS-CoV-2

#### 3.3.1. Cruise Details

Fifteen studies reported different aspects of the SARS-CoV-2 outbreak from the DP [7,17,19,20,21,23,24,25,26,27,29,30,31,35,37]. Six studies detailed the ship’s technical specifications [23,25,26,30,31,35], focusing mainly on cabin capacity and occupancy, and distribution of cases according to different decks. One study also mentioned that internal air recirculation was stopped in the ship (from day 16 onwards) [30].

The DP departed from Yokohama on 20 January 2020 and visited six ports in three countries (Japan, Hong Kong, and Vietnam). The timeline of the cruise is presented in Supplementary Materials File S7. However, only one report presented a detailed itinerary [30], and group activities were investigated by three studies [26,30,35]. On 3 February, the ship returned to Yokohama. On 5 February, passengers were quarantined in their cabins for 14 days; the crew continued to maintain the ship’s functions and assist passengers with food, clothing, and shelter-related needs.

On 19 February, the disembarkation of uninfected passengers began. Forty-one days after the ship started the voyage, on 1 March, disembarkation was completed, with the last crew members leaving the ship [7].

The total number of people onboard the DP was reported to be 3711 [7,19,21,25,26,27,31,35,37] or 3713 [30]. The number of passengers was reported to be 2666 [7,19,25,27,31] or 2645 [30]. The number of crew was reported to be 1045 [7,19,25,27,31] or 1068 [23,30]. Four studies did not report the number of people onboard [17,20,24,29]. In addition, one study on SARS-CoV-2 outbreaks on international ships reported 3200 passengers on the DP [6].

Nine studies reported SARS-CoV-2 outbreaks at the international level [6,7,18,22,28,32,33,34,36]. Nonetheless, the total number of the voyages could not be calculated, as some authors did not report specific data about the cruise (i.e., names of the ships or origin and destinations of vessels). Therefore, some voyages may have been included in several different reports. The total number of passengers and crew members in each study is presented in Table 1.

One study presented data on 89 voyages, including 70 ships from US waters or carrying US citizens; 16 ships had recurrent outbreaks [18]. Other authors [6] reported an analysis of 36 ships with COVID-19 globally and an analysis of data from 10 cruise liners with SARS-CoV-2 cases from Australia.

One study reported COVID-19 cases from two Nile River cruises [28], but the total number of passengers and crew was not mentioned. Two studies reported on single cruise ship outbreaks (Greg Mortimer ship [22] and Grand Princess [7]). Two studies reported on the outbreak on the Costa Atlantica cruise ship, docked at Nagasaki City since January 2020 for complete maintenance, with only crew on board and no passengers [32,36]. Another report investigated the SARS-CoV-2 outbreak on a Norwegian cruise vessel [34], with two one-week voyages, and another study analyzed data on five cruises with 80 itineraries between the different Canary Islands [33].

The technical specifications of the ship were provided in one study [33]. Four studies reported the voyage duration [22,28,34,36], and the itinerary was detailed by one study [22]. All studies were conducted in the first half of 2020, except the Norwegian cruises (July–-August 2020) [34] and the cruises in the Canary Islands (November 2020–May 2021) [33]. The latter study was performed after the implementation of vaccination programs. However, none of the COVID-19 cases were vaccinated, and information regarding the vaccination statuses of other passengers and the crew was unavailable to the authors [33].

#### 3.3.2. Case Definitions: Index Cases, Contacts, and Secondary Cases

The definition of index cases, contacts, and secondary cases varied across studies (Supplementary Materials File S8). Eight studies reporting the DP outbreak [17,19,20,21,24,29,35,37], and 10/11 of the studies reporting other international outbreaks [6,7,18,22,28,32,33,36] did not provide a clear definition of the index case. In addition, close contacts were considered to be cabinmates of confirmed case-patients [25,30] or suspected case-patients [30], individuals who had been in a room with someone for more than 15 min without wearing a mask [26,33], or passengers who joined the Kagoshima tour with the index case from the DP [30]. Case definitions for secondary infections included asymptomatic and symptomatic passengers or crew.

#### 3.3.3. Study Types and Contact Tracing Strategies

Most studies presented a retrospective follow-up of passengers and crew after identifying one or more cases of SARS-CoV-2 infection (Table 1, Supplementary Materials File S8). Nine studies reported an active case-finding [22,25,30,31,32,33,34,35,36], and six studies presented a comprehensive follow-up strategy [22,25,31,32,34,36]. Some authors also used travel information from the ship manifest [25], cruise ship company [36], questionnaires [7,26,35], surveys [23], smartphone-based remote health monitoring system [36], or data from different databases [6,18,19,29,35,37].

On the DP, initially, only individuals with fever or respiratory symptoms and their close contacts were tested by RT-PCR for SARS-CoV-2. Subsequently, to support phased disembarkation of passengers, testing was expanded, prioritizing older individuals, people with comorbidities, and people accommodated in interior cabins without access to the outdoors [7,25,31].

Eight studies followed up only a sub-population: repatriated cases [17,21,26,29,35], crew [23], or individuals admitted to a single center [20,24]. Considering all the studies, the proportion of traced contacts ranged from 0.19 to 100% in these studies. The total numbers of identified passengers and crew and the total number of successfully traced individuals could not be calculated as several studies did not report specific data on the number of passengers, crew, or medical staff on board [6,18,28] (Table 1).

### 3.4. Onboard Transmission

#### 3.4.1. Reporting of Index Cases, Secondary Cases, Contact Tracing, and Follow-Up

Four studies on the DP outbreak did not provide any information on the index case; the remaining eleven studies report inconsistent data. Several authors considered the index case to be an 80-year-old man who boarded the ship on 20 January and disembarked in Hong Kong on 25 January [7,19,21,26,27,30,35]. However, another study reports that a different passenger with laboratory-confirmed COVID-19 developed symptoms on 22 January [23]. Other authors considered the case reported by Hong Kong authorities merely an indicator case, i.e., the first detected individual among many infected persons [25]. Another study suggested that the outbreak most likely originated from either a single infected person, or simultaneously with another primary case [37]. Additionally, data on both the nature and, most importantly, the onset of symptoms was highly variable. Yamagishi et al. reported that the Hong Kong passenger had a cough starting one day before embarkation [30]. Other authors reported that the index case presented with a mild cough on 23 January [19,27], and developed a fever on 29 January [19] or 30 January [31]. Many cases had onset dates before the ship arrived in Yokohama and, by that time, infection had already spread across several decks without any spatial clustering [25].

Five studies reporting SARS-CoV-2 outbreaks on over 100 international ships did not present any data on index cases [6,18,28]. In five studies, the number of index cases could not be determined [7,22,33,34,36], as multiple passengers or crew presented with respiratory symptoms around the same period. In 11 studies with data on the index case, the laboratory diagnosis was based on binary RT-PCR results, without data on Ct (Supplementary Material S8). Two studies provided the Ct cutoff value [32,34]. The Costa Atlantica crew was initially assessed with a reverse transcription loop-mediated isothermal amplification (RT-LAMP) assay; RT-qPCR confirmed the results of the COVID-19-positive samples [32,36]. The timing of RT-PCR is clearly stated for the Hong Kong passenger from the DP and for passengers and crew from two other reports [7,22]. On the Greg Mortimer ship, the tests were performed on the 20th day of the voyage, 12 days after the first case presented symptoms [22]; on the Grand Princess, the RT-PCR was performed on the 23rd day of the voyage [7].

On the DP, by the end of the quarantine period on 20 February, 619 cases (537 passengers, 82 crew members) had been confirmed [25]. By 8 March, 696 secondary cases were reported [20,25,31]. Additional cases were found after the repatriation of Hong Kong [21], US [26,29], Australian [35], and Israeli citizens [17]. A total of 712 individuals had tested positive as of August 2020 [19].

The secondary cases on the DP were reported as asymptomatic, pre-symptomatic, and symptomatic. At that time, only respiratory symptoms and fever were considered indicative of SARS-CoV-2 infection. The studies with a comprehensive tracing strategy found an attack rate (AR) of 18.5 [25], 18.8 [31], or 19.2% [7]. The number of asymptomatic cases at the time of testing, based on the limited definitions for symptomatic illness in use at the time, was reported to be 46.5 [7], 55.0 [25], and 58.9% [31]. Up to 24 February, among 687/3711 individuals with SARS-CoV-2 infection, 544 (20.4%) were passengers, and 314 (57.7%) of those were reported to be asymptomatic. In addition, 143 (13.7%) were crew members, with 64 (44.8%) reported as being asymptomatic. Symptomatic cases among passengers peaked on 7 February. Thirty-five passenger cases had symptom onset before 5 February. Cases among crew members peaked on 11 and 13 February [25].

Sekizuka et al. [27] reported detailed information on the RT-PCR results of 896/3711 individuals from the DP, with 148 (16.5%) positive results (Cq 16.00–38.31). Among 65 symptomatic individuals, 22 had a positive RT-PCR. In addition, 125/830 (15.1%) asymptomatic cases had a positive RT-PCR [27]. Symptoms were not reported for one case with a positive RT-PCR [27].

In crew members, the earliest case of infection was detected in a food service worker who developed a fever on 2 February and had a positive RT-PCR the following day [23]. By 9 February, 31 crew members reported with a fever, but only 20 had a positive RT-PCR test; 15/20 confirmed cases were food service workers, and 16/20 cases occurred among persons with cabins on deck 3, where food service workers lived [23].

One study investigating SARS-CoV-2-positive crew members from the DP found that 4/7 cases presented with throat swab cultures and sputum samples that were positive for bacterial infections (*Haemophilus influenzae*, *Klebsiella pneumoniae*, and *Staphylococcus aureus*). Three cases were asymptomatic and four presented with a cough [20]. Also, among Australian citizens, five individuals were confirmed positive for Influenza A [35].

In Hong Kong repatriated citizens, eight cases that tested negative in Japan had a positive RT-qPCR upon arrival, five days later [21]. The median viral load in nasopharyngeal samples at baseline was 4.31 log10 copies/mL (IQR 3.79–6.65). One case presented anti-RBD IgG with undetectable viral load. Six patients remained asymptomatic during quarantine [21]. In Israeli repatriated citizens, 4/6 confirmed cases were initially diagnosed in Japan and 2/6 upon arrival to Israel (with Ct values of 24–40) [17]. Three patients were reported as being asymptomatic, and others were paucisymptomatic [17]. In the US repatriated citizens [7,26,29] (Supplementary Material Files S6 and S8), the most comprehensive study [26] reported that 114/437 (26%) individuals tested positive for SARS-CoV-2; 98 cases had a positive RT-PCR in Japan, 10 cases tested negative in Japan but had a positive result in the US, and 6 cases were never tested in Japan, with a positive result in the US [26]. Of 66 travelers with positive tests and complete symptom information, 14 (21%) were asymptomatic while on the ship [26]. Among Australian citizens, 46/56 were diagnosed in Japan and 10/56 after repatriation, with 29% asymptomatic cases [35].

On other international voyages, the number of secondary cases were not reported [6,18,36] or were unclear [7,22,33,34,36] because the number of index cases were unknown. The authors reported only the total number of passengers or crew infected with SARS-CoV-2. One GS study, reporting on two Nile River cruises, found three passengers on one cruise and two on another, all symptomatic, who were most probably infected during their voyage [28]. The authors traced only these passengers, without any data on other passengers or crew [28]. The AR varied between 7.67 and 75.12% [6,7,18,22]. The analysis of 36 cruise ships with reported SARS-CoV-2 infections worldwide showed an overall AR of 8.66% (0.03–75.10%) [6].

#### 3.4.2. Spatial Distribution

Spatial distribution of passengers and crew was investigated in eight studies on the DP [7,23,25,26,27,30,31,35] and seven studies on international ships [6,7,22,32,33,34,36]. On the DP, >80% of crew cabins were on decks 2–4. Initially, most cases occurred among persons with cabins on deck 3 [23]. By the end of quarantine, the distribution of infections from the crew decks produced a more generally distributed pattern, but a large number of cases were noted on deck 3 [25]. The mean number of persons per cabin was 1.73 (1 to 3) for crew and 1.98 (1 to 4) for passengers [7].

Another study from the DP conducted between 3–9 February reported that the ARs among passengers were similar across decks. Among confirmed cases, 144 (84%) met the definition of a suspected case before testing, whereas 19 (11%) shared a cabin with a confirmed person. There were 24 asymptomatic cases (14%); most of them were passengers who joined the Kagoshima bus tour (with the assumed Hong Kong index case) [30].

Among DP passengers, ARs were highest in those who stayed in four-person cabins (30.0%; *n* = 18), followed by three-person cabins (22.0%; *n* = 7 cabins), two-person cabins (20.6%; *n* = 491 cabins), and single-person cabins (8%; *n* = 6 cabins) [25], suggesting a distinct dose–response relationship. The infected passengers were distributed across different decks without aggregation or large-scale clustering by deck or zone [25,27].

In US citizens [26], the AR for passengers in single cabins or without infected cabin mates was 18% (58/329), 63% (27/43) for passengers that shared the cabin with an asymptomatic infected person, and 81% (25/31) for those with a symptomatic infected cabinmate, again suggesting a dose–response relationship (*p* < 0.01). Genome sequences from persons sharing cabins clustered together [26]. In addition, the association between attending some events, such as the bus excursion in Cai Rang, and group activities on 3–4 February, suggested several everyday mass exposure events [26].

In Australian citizens, before the quarantine, exposure to a close contact or cabinmate confirmed later as SARS-CoV-2 positive was associated with a 3.78-fold (95%CI, 2.24–6.37) higher risk of infection. Exposure to a positive cabinmate during the quarantine period resulted in a significantly increased risk of infection RR 6.18 (95% CI, 1.96–19.46) [35]. The authors found no statistically significant association between participation in shore trips, tours, or social events before quarantine or visiting public areas during quarantine and subsequent infection [35].

Other authors also noted that during quarantine on the DP, the elevator hall situated in front of the medical center may have presented a higher infection risk because infected and uninfected people could not use the elevator separately [31].

Studies on other international ships also reported on the spatial distribution of people on board. On the Greg Mortimer, in 10 instances, two passengers sharing a cabin presented positive and negative results [22]. On the Grand Princess, the median number of persons per cabin was 1.95 (range of 1–4) for passengers and 1.75 (range of 1–4) for the crew; among 469 persons with available results, 78 (16.6%) had a positive RT-PCR [7].

A study investigating 36 cruise ships [6] found that the number of available cabins presented a moderate inverse correlation with the AR; as the number of available cabins per ship increased, the AR decreased. There was a fair inverse correlation of decks with cabins; the more spread out the cabins were through several decks, the lower the AR. Also, the authors found a moderate positive correlation with the passenger-to-space ratio; as the passenger-to-space ratio increased (i.e., the ship became more crowded), the AR increased [6], suggesting a dose–response relationship. The AR was predicted by all spatial distribution variables (i.e., number of cabins per ship and decks with cabins), but not by cruise duration [6].

In the Canary Islands cruises, with a preventive protocol, the AR between the close contacts during quarantine was 3%, and 21% of confirmed cases were casual contacts of a case at onboard food and beverage venues [33].

Another study reported on a ship with updated but not fully implemented prevention protocols. The first symptomatic cases worked in catering and mechanical operations. Nine days later, staff working in administrative and passenger service areas reported symptoms. There were no cases among participants working in electricity, carpentry, or medical and spa services. The authors note that the risk of SARS-CoV-2 infection was associated with working in mechanical operations (OR 8.26, 95% CI 1.54–44.16) and catering services (6.06, 1.78–20.67). Sharing a cabin with an infected case was significantly associated with an increased risk of infection in crude analysis (7.20, 2.48–20.41), but was found to be non-significant in the full model (3.27, 0.97–11.07) [34].

On the Costa Atlantica ship, the infection was probably introduced by a crew member from the entertainment occupation group, which then spread widely inside the vessel, regardless of occupational group or location of the crew cabin. The cases were similarly distributed across occupation types. Also, the cabin rooms of crewmembers presenting with a fever were widely distributed throughout the ship [36].

#### 3.4.3. Use of Masks

Three studies reported the use of masks on the DP after quarantine started [23,25,30]. The crew used surgical/N95 masks [30]. None of the authors specified if a “fit test” was performed to evaluate if the mask fit and sealed properly.

Organized by deck and section, passengers were allowed a 60-min daily period on an exterior deck. During this time, they were required to wear masks, not touch anything, and maintain a one-meter distance from other people. Meanwhile, they were observed by monitors. After each group, the areas were disinfected [25]. Ten studies do not provide any information on masking [7,17,19,20,21,24,26,27,29,31]. On international ships, one study reported using masks after the quarantine was issued [22]. Also, masks were required for all people onboard indoors, in spaces of shared use (including excursions), except when eating, drinking, or staying in the cabins [33].

Alternative exposures were not fully evaluated in 8/9 studies on international ships [6,7,18,28,32,33,34,36] and four studies on the DP [17,19,29,35].

### 3.5. Genome Sequencing (GS) and Phylogenetic Analysis

Five studies on the DP outbreak [19,24,26,27,37] and one on the passengers from the Nile River cruises [28] performed GS and phylogenetic analysis. The methods used for performing these investigations were essentially similar across studies (Supplementary Material File S9).

Sekizuka et al. [27] analyzed 70 whole-genome sequences obtained from RT-qPCR positive samples. These sequences and three additional DP isolates from the Global Initiative on Sharing All Influenza Data (GISAID) were compared with the Wuhan-Hu-1 genome sequence. The frequencies of single nucleotide variations (SNVs) indicated that all 73 isolates shared an SNV (52 SNVs in DP isolates vs. 449 SNVs in all the isolates, including GISAID entries) [27] (Supplementary Material File S9).

The DP-A cluster was predominant (29 isolates), suggesting that it was the ancestral haplotype for subsequent transmission. Although further spreading may have been prevented by quarantine, some of the subsequent progeny clusters, as well as DP-B (five isolates) and DP-C (six isolates), were probably formed via transmission through other links, such as mass gatherings in recreational areas and direct transmission among cabinmates. In addition, 33 patients (45%) not included in the DP-A, -B, or -C clusters had unique SARS-CoV-2 haplotypes and patient-specific unique SNVs and/or deletions [27], suggesting there may have been multiple introductions of different strains on the DP. All whole-genome sequence data were deposited to GISAID [27].

Murata et al. [24] performed GS of four sequential specimens collected from one infected person (Carrier_1) and nine specimens obtained from his cabinmate and six others. All SARS-CoV-2 strains belonged to clade 19A, with a single nucleotide mutation (G11083T transversion), as previously described [27], suggesting this strain was transmitted between these cabinmates and the six others. The GS analysis of consecutive samples of Carrier_1, who shed infectious virus for 15 days, identified the emergence of two novel SNVs (C8626T transition and C18452T transition) in the sample collected on day 15. None of these mutations were found in samples collected from the cabinmate of Carrier_1 and other cases [24].

Plucinski et al. [26] reported data on GS from samples obtained from 28 individuals that tested positive after repatriation. All genome sequences clustered in the B group of the global phylogenetic tree, containing all the genome sequences reported previously on the DP. [26]. All genome sequences presented the same mutation reported in the assumed Hong Kong index case [26].

Twelve sequences were from six pairs of close contacts. In all instances, pairs of linked genomes grouped closely within the haplotype network. The linked sequences were separated by zero to two SNVs compared with zero to nine SNVs among all sequences from the DP [26].

Another study [19] retrieved all publicly available SARS-CoV-2 genome sequences with clinical information from the GISAID database up to 7 August 2020 (*n* = 78,448). The phylodynamic analysis of 67 sequences collected between 10–17 February 2020 estimated that the outbreak originated on 21 January, coinciding with the boarding of the presumed index case from Hong Kong. The affected population size increased around 30 January and exponentially surged from 2 February, before the quarantine. After quarantine, the transmission of the virus slowly continued [19]. Although branch bootstrap values were low, all sequences from the DP clustered with some isolates reported in other countries [19].

Yeh et al. [37] analyzed the evolution dynamics of SARS-CoV-2 in 28 cases from the DP. They identified 24 new viral mutations across 64.2% (18/28) of samples; the virus evolved into at least five subgroups. Based on their findings on the limited number of cases they analyzed, these authors suggested that the outbreak most likely originated from either a single person or simultaneously with another primary case infected with a virus containing the G11083T mutation [37].

On the Costa Atlantica ship, the authors analyzed all samples with high viral titers (Ct < 30 by RT-qPCR), obtaining 94 complete GS [32]. The strains showed three main clusters; the CA-A cluster was genetically closest to the reference strain (Wuhan-Hu-1), possibly indicating that it was the haplotype initially introduced into the CA cluster. CA-A was not a large cluster; only two infected persons perhaps had a central role in spreading SARS-CoV-2 in the CA-A cluster. The core populations of clusters CA-B and CA-C comprised of more than ten individuals, indicating that superspreading event-like infections caused these clusters [32].

Sekizuka et al. [28] reported data on GS from five SARS-CoV-2-positive passengers from two Nile River cruises. Three passengers aboard the same ship presented identical SARS-CoV-2 genome sequences, with a close lineage to isolates from Europe. In addition, a couple of passengers boarded a different cruise ship, but had the same Tokyo to Cairo flight as one of the travelers reported above. These two SARS-CoV-2 isolates showed identical GS, but differed from the genome sequences of the first three travelers by only one SNV [28]. The authors compared the GS of the passengers with the only two genome sequences of SARS-CoV-2 isolates in Egypt available in GISAID at the time of the study. The haplotype network exhibited that one of the first cruise passengers and the two passengers from the second cruise was closely related to isolates from Egypt, with only two or three differences in SNVs [28].

### 3.6. Viral Cultures

Two studies performed viral cultures [17,24] (Table 1; Supplementary Material Files S8 and S9). Murata et al. [24] analyzed 116 PCR-positive samples and 50 PCR-negative samples. The median Ct value of culture-positive samples was 24.6 (IQR, 20.4–25.8; range, 17.9–30.3) vs. culture-negative samples (Ct 35.9). SARS-CoV-2 was successfully cultured from nine (7.8%) PCR-positive samples obtained from seven carriers; none of the PCR-negative samples presented cytopathic effects (CPE) [24]. A specimen from a 70-year-old woman with a medical history of diabetes mellitus and hypertension, who had prolonged RT-PCR positivity for >21 days, was found to have CPE on culture after 15 days. This was confirmed using PCR, following the initial positive PCR test [24], but the result may be questionable given the Ct values and passage of time.

The second study [17] reported on cell cultures of a 62-year-old woman with comorbidities. She presented with two negative RT-PCR tests before repatriation, but tested positive upon arrival to Israel (Ct = 24). The nasal and throat swabs sampled four days later showed a notable CPE on Vero E6 cell culture, but no data was provided on the methods used for cultures [17].

## 4. Discussion

### 4.1. Summary of Main Findings

We identified 23 studies assessing SARS-CoV-2 transmission aboard cruise ships, two environmental studies, and one systematic review. The findings suggested lower ARs in ships with a higher number of available cabins, with cabins more spread out over various decks, and in settings with a lower passenger-to-space ratio. However, the duration at sea did not appear to influence the AR [6]. In addition, a consistent dose–response relationship was found in multiple studies, demonstrating that as the number of passengers in a cabin decreased, ARs decreased [25,26,35], with the highest ARs in individuals staying in four-person cabins (30–63%) and the lowest (8–18%) in single-person cabins [25] or without infected cabinmates [26]. The AR for those sharing a cabin with an asymptomatic infected cabinmate was lower compared with passengers with a symptomatic infected cabinmate [26]. The risk of infection was higher if an individual had close contact with a confirmed case [35]. Environmental samples found no difference in detection proportion between cabins of symptomatic and asymptomatic cases [16], but there were only a limited number of samples and they were taken after the ship was vacated, making the interpretation of the environmental positives uncertain. A potential common exposure area, with higher infection risk, was suggested to be the elevator hall [31].

Participation in events such as excursions or other group activities was associated with an increased risk of infection [26,30], with the exception of one study that found no statistically significant association in this regard [35].

Epidemiological studies from the DP suggested that passengers and crew members presented symptoms from the beginning of the cruise; the number of suspected cases (with symptoms or close contacts) remained low for two weeks, followed by a notable increase [30]. The infected passengers were distributed across different decks, without any identifiable aggregation or large-scale clustering by deck or zone [25]. The effective population size began to increase around the 10th day of the voyage, surging exponentially from the 13th day. After quarantine, the infection transmission continued more slowly, based on one interpretation [19]. The first crew member that tested positive from the DP presented symptoms ten days after the first passenger [23,30]. Most crew infections were among food service workers [23], with the highest AR on the deck where most food service workers lived, suggesting that infection spread during ship activities such as parties [23,30].

The role of masks for preventing SARS-CoV-2 transmission aboard cruise ships remains unclear. On the DP, masks were used after the start of quarantine [23,25,30], with ARs reported between 18.8 [31] and 19.18% [26]. On international ships, one study reported that use of masks was associated with an AR of 59% [22]. With the implementation of mitigating measures and pre-disembarkation screening, ARs between the identified close contacts during quarantine was 3% [33]. Lower ARs (i.e., 0.03%) and higher ARs (i.e., 75.12%) were also reported on some ships [6], but the role of masks as a mitigating measure was not assessed. On the Costa Atlantica, with only crew on board, the AR was 24% [36]. When probable cases were included (with symptoms indicative of COVID-19 but a negative test result), the AR was 41% [36].

Researchers reported the possibility of virus transmission from asymptomatic, pre-symptomatic, or symptomatic individuals. Nonetheless, a significant limitation of all studies was the possibility of “asymptomatic” index cases transmitting the infection and “asymptomatic” secondary cases not being investigated due to a lack of fever or respiratory symptoms. Although these are part of the COVID-19 symptom complex, using these symptoms alone would have decreased sensitivity and grossly overestimated the number of asymptomatic cases. In addition, failure to identify a common starting time for exposure or illness may have led to systematic misclassification and failure to identify the existence of multiple index cases leading to biased AR estimates. Furthermore, the number of studies reporting Ct of RT-PCR was limited; therefore, case ascertainments are likely biased [14]. Also, the timeline of sample collections after disembarking may have led to bias if there were contacts with others after leaving the ship.

The GS studies [19,24,26,27,28,32] reported high-quality evidence supporting transmission of SARS-CoV-2 on cruise ships. Some studies suggested that, on the DP, there was a single starting source of infection with the virus bearing a G11083T transversion mutation. They also provided evidence of zero to two mutations per genome being the norm when comparing the virus in the putative source with those in the target cases. Although Hoshino et al. [19] found most isolates were very similar, based on only a two to three SNV difference, some strains clustered with strains from other countries, so it was difficult to rule out introductions of other strains from other passengers. In addition, although Sekizuka et al. [27] reported all their analyzed strains had the G11083T mutation, there were up to 449 SNV differences between all isolates, and 33 patients had uniquely different sequences despite having the mutation, making it difficult to rule out differing virus strain introductions. Whether there was a single or multiple isolate entries to the DP from multiple passengers remains uncertain, but the weight of all the evidence supports multiple entries [23,37]. Also, the number of mutations in the DP cluster was remarkably lower than that of the Costa Atlantica cluster, indicating that there were some environmental differences between the two cruise ships [32]. In addition, although GS methods may provide reliable phylogenetic insights into the relationship between the putative index and secondary cases, using GS databases to ascertain transmission may induce bias if the number of published sequences is limited [39]. Missing data may also induce bias in phylogenetic analyses, and substantial gaps in global sequencing data may hinder the accurate recognition of an infection source.

The positive results of viral cultures [17,24] provide further evidence of transmission of SARS-CoV-2 aboard cruise ships, indicating an infectious virus with the potential for transmission to other individuals was present. The chain of transmission to secondary cases was well-documented by evidence confirming that the index case was contaminated (i.e., low Ct values) with an infectious virus and confirmed by GS. In the environmental study, no viable virus could be isolated [16]. However, the high Cq values in most positive samples likely explain the negative viral cultures [16]. Viral cultures were not performed on the index cases [17,24], but they provided important insights on SARS-CoV-2 transmission. Murata et al. [24] demonstrated “asymptomatic” carriers on the DP [40].

The possibility of alternative exposures deserves attention. Common sites of alternative exposures include sites before embarkation (i.e., waiting lines), during the cruise (i.e., tours), and after disembarkation (i.e., lining up to exit the ship, checking documents, and traveling to the final destination).

One study found that 4/7 crew members from the DP presented with bacterial co-infections [20]. Also, another study investigating 896 RT-PCR samples from the DP reported that 43/65 (66.15%) symptomatic cases had a negative RT-PCR [27]. Nonetheless, only two studies [20,35] presented data on investigations aiming to detect a possible co-infection in SARS-CoV-2 positive cases or ran any additional laboratory tests to investigate the cause of acute respiratory illness (ARI) in individuals with a negative RT-PCR. In Australian citizens [35], ARI was reported as 62/196 (31.6%) for those with a negative SARS-CoV-2 test, and 52/62 (84%) of these individuals could identify their onset date; 37 individuals reported symptom-onset dates on board whereas 15 reported symptom-onset dates after quarantine in Australia. Five individuals were Influenza A positive, including one case who tested Influenza-A-positive and a fortnight later SARS-CoV-2-positive. ARI symptoms were reported starting from the first day on the DP in three passengers. Seventy-one percent (25/35) of symptomatic COVID-19 cases reported experiencing fever (≥37.5 °C), in contrast to 23% (14/62) of individuals with non-specific ARI (*p* < 0.001) [35]. ARI symptoms may be present in COVID-19 cases, but they are also found in other viral or bacterial infections, including Legionellosis. Furthermore, Legionella co-infection has been reported in cruise ship passengers with COVID-19 [41]. ARI accounted for up to 29% of recorded illnesses on cruise ships [3,42], and in many cases, it was due to respiratory viral infections [43,44]. One surveillance project reported that 83% of crew members and passengers with ARI tested positive for at least one respiratory virus; 71% had Influenza A or B virus. Over three years, 13 respiratory viruses were identified, including influenza, human rhinovirus, human metapneumovirus, parainfluenza, and adenovirus C, with nine different co-infections [44]. In COVID-19 patients, an undetected co-infection could lead to biased results in fatality rate or hospitalization. Also, excluding other pathogens as a cause of symptoms is important. An incomplete investigation may cause mimicry bias, leading to false conclusions about the causes of the disease of interest.

Further doubts about the validity of the overall findings are raised by the variability in contact tracing strategies, contact tracing timelines, proportion of passengers and crew that were traced successfully, use of distinct case definitions, testing strategy, and case ascertainment.

Only one study in the present review showed evidence of both positive virus cultures as well as genomic evidence [24]. Definitive route(s) of transmission on cruise ships need further investigation. On the DP, the possibility of long-range airborne transmission could not be ruled out at the time of the initial outbreak, but is now considered unlikely given the dose–response gradient with higher numbers among cabinmates and the fact that internal air recirculation was stopped [30]. Evidence from the available studies suggests that transmission on the DP was associated with close proximity [35] and potentially with common source exposure events [25,30]. More recent viral culture research reports significant amounts of infectious SARS-CoV-2 in the environment [45,46,47]. Although no live virus was found on cruise ships on fomites, a recent systematic review focusing on high-quality studies found that replication-competent SARS-CoV-2 was present on fomites [47]. Replication-competent SARS-CoV-2 is significantly more likely when the PCR Ct for clinical specimens and fomite samples is <30. However, the timing of sample collection from symptom onset markedly influences the probability of obtaining positive viral culture results [47].

Our review did not compare risks between cruise ships and other similar settings (e.g., other types of ships). However, because cruise ships often have high-risk senior passengers and may offer comprehensive medical services (e.g., oxygen therapy and dialysis units), they could present infection control problems similar with those in nursing homes. Multiple studies have demonstrated very high transmission rates of other viruses on cruise ships including norovirus and other respiratory viruses [48,49].

To date, only one systematic review assessed the evidence for transmission of SARS-CoV-2 aboard ships [38]. However, the literature search went up until July 2020. The authors included 37 studies on cruise, navy, and cargo ships, but many of the included studies were not relevant for SARS-CoV-2 transmission (i.e., case reports on conjunctivitis or clinical aspects of pneumonia among ship passengers or crew). Furthermore, they did not include environmental studies and did not formally assess study quality.

### 4.2. Strengths and Limitations of the Review

We performed an extensive literature search, accounted for the quality of included studies, and reported all relevant outcomes, including GS and viral cultures. We included results from one non-peer-reviewed study [29], which may affect the reliability of the results. However, due to the ongoing pandemic, such studies could potentially be of research benefit. Our review is one of the most comprehensive ones to date. By focusing on higher-quality studies, we were able to draw meaningful interpretations, which facilitates our understanding of the transmission aboard cruise ships.

Limitations of the present review are mostly related to the low quality of included studies and the fact that different studies provided different data on the same outbreak with inconsistent results. In addition, data extraction was challenging due to missing, incomplete, or unclear descriptions of investigations. Also, most primary studies investigated only some aspects of the outbreak (e.g., spatial distribution, a subgroup of individuals from a cruise, or the early days of the outbreak).

Various reasons may explain the low quality of the published literature. There is a lack of standardized methodology and clear reporting criteria, with substantial methodological variation in SARS-CoV-2 transmission studies [40]. Nonetheless, similar to other studies on SARS-CoV-2 transmission in other closed/semi-closed settings, in times of a pandemic, the opportunities for rigorous studies that trace, interview, and test hundreds or thousands of individuals are challenging and often lacking [50,51]. The likelihood of case ascertainment bias based on symptoms was likely higher at the time of the initial cruise ship outbreaks, given that the full symptom complex of SARS-CoV-2 was underappreciated. Other types of bias were also discussed in previous paragraphs.

That there was a risk of SARS-CoV-2 transmission on cruise ships was evident a few weeks after the DP outbreak. Nonetheless, 2.5 years later, we still lack definitive information on cruise ship transmission modes. Furthermore, only one study was performed after implementing vaccination programs; however, none of the COVID-19 cases were vaccinated [33]. Information about the vaccination status of the rest of the passengers and crew were not available to the authors [33]. We found no studies on more recent variants such as Delta or Omicron.

Therefore, besides its historical value, the present review raises awareness of the paucity of data regarding this topic and the necessity for high-quality research on future cruise ship outbreaks. Otherwise, we will be unable to understand and prevent any similar disease outbreaks with Omicron variants, other future SARS-CoV-2 variants, or other respiratory pathogens.

We did not include lists of public health authorities on SARS-CoV-2 transmission aboard cruise ships. However, we included studies reporting analyses of public lists with retrospectively known cases of SARS-CoV-2 infection aboard ships.

### 4.3. Implications for Practice and Research

Our findings highlight the need for a standardized approach to investigate and report SARS-CoV-2 transmission aboard cruise ships, with possibly a standard international protocol to investigate ship-borne outbreaks. Future research should aim for a thorough epidemiological investigation, a comprehensive evaluation of passengers and crew, with a comprehensive symptom and signs assessment, a rigorous follow-up strategy, and a more robust testing strategy. Factors that may influence transmission should be consistently assessed: pre-embarkation screening strategies; technical specifications of the ship; voyage duration and the number of ports of call; movement and activities (i.e., tours, social activities, drinking or eating, contact with contaminated surfaces, and use of elevators or lavatory in common areas); passenger and crew spacing; onboard screening/surveillance procedures; infectivity of the index case (asymptomatic, pre-symptomatic, or symptomatic, vaccination and immunological status, and mask-wearing or not); the susceptibility of passengers and crew (previous SARS-CoV-2 infection or vaccination and compliance with masks and distancing); and effectiveness of exposure (proximity to the index case and exposure duration).

Future studies should provide Ct values when reporting RT-PCR results and present data on timing and methods of sample collection. Further research, including virus isolation, GS, and phylogenetic analysis, should be conducted to strengthen the current evidence. Consequently, standardization of research reporting should be a priority.

Cruise ships accommodate large numbers of passengers and crew members originating from different countries. In addition, passengers are often older with multiple comorbidities and an increased risk for severe disease and complications. Also, the close quarters, partially enclosed settings, and prolonged contact among individuals increase the risk of infectious disease transmission. The presence of other viral and bacterial causes of ARI in the context of a SARS-CoV-2 outbreak adds additional complexity because COVID-19 may present with similar symptoms. Methods for assessing disease conditions that mimic the inciting cause should be clearly defined, including testing strategies, procedures of isolation, notifications of the authorities, and criteria for returning to work [43]. An integrated syndromic and virologic surveillance could improve the detection and characterization of SARS-CoV-2 outbreaks and other respiratory pathogens [44]. Although expensive, adding a laboratory component to routine cruise ship respiratory surveillance could inform better resource allocation and anticipate needs for cruise ship populations. In addition, it should be considered that some passengers may not have international or travel healthcare insurance coverage. Therefore, costs could restrain passengers from seeking medical care until they present with severe illness. Surveillance of only symptomatic passengers or crew reporting to the ship’s infirmary may bias detection of cases to those with more severe symptomatology or those more likely to seek healthcare [44].

Moreover, a SARS-CoV-2 outbreak on a cruise ship has substantial economic and human resource costs for both public health agencies and cruise ships. Additional staff may be needed to implement active and passive surveillance and organize testing; during illness or isolation, crew time is lost, and treatment and hospitalization costs can be expensive. Therefore, the potential benefits should be weighed against the operational limitations of a thorough surveillance program.

Several mitigating measures were introduced to prevent the transmission of SARS-CoV-2 aboard cruise ships. However, the zero-COVID-19 countermeasures presented serious fiscal consequences, and a more practical near zero-risk approach was recently proposed [52]. The latest point of view advocates a holistic mitigating perspective, including behavioral (i.e., social distancing), procedural (i.e., different boarding times), and technical (i.e., testing procedures) measures.

The measures taken at the beginning of the pandemic reflect the difficulties of implementing zero-risk countermeasures, including high costs and logistics. In addition, to implement efficient mitigating measures (e.g., respiratory isolation processes PPE, airflow. Etc.), we first need a thorough understanding of transmission routes and risk factors. Therefore, high-quality research, with at least one study per type of setting and intervention, is required.

## 5. Conclusions

Current evidence indicates a definite risk of transmission of SARS-CoV-2 aboard cruise ships, with crowding and multiple persons per cabin being associated with an increased transmission risk. The highest ARs were found in individuals staying in four-person cabins and the lowest in single-person cabins or those without infected cabinmates. However, the currently published data do not allow a conclusive assessment of the risk factors and extent of the transmission. Nonetheless, valuable information may be gleaned from the highest quality studies. We found that the quality of evidence from most published studies was low. The analysis of findings across studies was restricted by variations in study design and methodology. Standardized guidelines for performing and reporting future cruise ship outbreaks should be developed.

## Figures and Tables

**Table 1:** Onboard transmission studies.

Study	Year	Country	Ship	No. of Passengers and Crew on Board	No. of Passengers and Crew with SARS-CoV-2	No. of Index Cases	No. of Passengers and Crew Traced (%)	No. of Secondary Cases Identified (%)	Attack Rate (%)	No. of Secondary Cases in Close Proximity (%)	No. of Secondary Cases Not in Close Proximity (%)	Strength of Evidence
Álvarez-León	2022	Spain	5 ships	103,500 px, 3228 crew	19 px, 1 crew	Up to 6	20 cases (19 px and 1 crew member) and 96 close contacts (68 px from among 63709 px and 28 crew out of 1420	Minimum 13 px, 1 crew	N/A	N/R	N/R	Diagnostic test—unclear.
Goldenfeld	2020	Israel	Diamond Princess	N/R	6/15 Israeli citizens	N/R	15 px	6 px	N/A	N/R	N/R	RT-PCR with Ct in 3 cases. Ct up to 40. Viral culture in 1 case (positive).
Hoshino	2021	Japan	Diamond Princess	2666 px; 1045 crew	712 (px and crew)	1 px	67 SARS-CoV-2 cases	711 (px and crew)	N/A	N/R	N/R	GS; phylodynamic analysis; RT-PCR; no data on Ct.
Hoshiyama	2020	Japan	Diamond Princess	N/R	696	N/R	7 crew	7 crew	N/A	N/R	N/R	RT-PCR, no data on Ct. Bacterial cultures for co-infection.
Hung	2020	Hong Kong	Diamond Princess	3711 (px and crew)	712 (px and crew)	1 px	215 px from Hong Kong	9 px	N/A	N/R	N/R	RT-PCR, with data on viral load, serology.
Kakimoto	2020	Japan	Diamond Princess	1068 crew	20 crew	The index case could not be determined.	1068 crew	20 crew	N/A	15/20 cases—food service workers; 16/20 cases—persons with cabins on deck 3, where the food service workers lived.	N/R	RT-PCR, no data on Ct.
Moriarty	2020	USA	Diamond Princess	3711 (2666 px; 1045 crew) including 428 USA px and crew)	712 px and crew	1 px	428 USA citizens	107/428 USA citizens	N/A	N/R	N/R	RT-PCR, no data on Ct.
Murata	2020	Japan	Diamond Princess	N/R	N/R	N/R	90 asymptomatic cases	90	N/A	N/A	N/A	RT-PCR, viral cultures, GS.
National Institute for Infectious Diseases Japan	2020	Japan	Diamond Princess	3711 (2666 px; 1045 crew)	696	Unclear. The “index case” was more probably an “indicator case”	3711	696	18.5%	AR among px were highest among those who stayed in 4-person cabins (30.0%; n = 18), followed by 3-person cabins (22.0%; n = 27), 2-person cabins (20.6%; n = 491), and 1-person cabins (8%; n = 6)	N/R	RT-PCR, no data on Ct.
Plucinski	2020	USA	Diamond Princess	3711 px and crew (437 US citizens)	712 px and crew	1 px	437 US citizens (including 229 survey respondents)	114/437 US citizens	26% (among US citizens)	Attack rates: from 17% in cabins without infected cabinmates to 81% in cabins with a symptomatic infected cabinmate.	N/R	RT-PCR, no data on Ct; GS (28 cases).
Sekizuka	2020	Japan	Diamond Princess	3711 (2666 px, 1045 crew)	697 px and crew	1 px	896 (880 px, 15 cre1, 1 quarantine officer) (24.11%)	148 cases (138 px 9 crew, 1 quarantine officer)	N/A	71	77	RT-PCR, with Cq, GS.
Walker	2021	Australia	Diamond Princess	3711 px and crew (223 Australian citizens)	712 px and crew	1 px	223 Australian citizens	56 Australian citizens	25% (among Australian citizens)	Attack rates: 1-person cabin: 0%; 2-person cabin —27%; 3-person cabin —6%; 4-person cabin —33%	N/R	RT-PCR, no data on Ct.
Waltenburg	2021	USA	Diamond Princess	N/R	N/R	N/R	328 USA citizens	45 USA citizens	N/A	N /R	N/R	RT-PCR, Ct < 40.
Yamagishi	2020	Japan	Diamond Princess	3713 (2645 px, 1068 crew)	172 (152 px, 20 crew)	1 px	490 (358 suspected cases, 86 close contacts)	172 (144 suspected cases, 19 close contacts)	N/A	19	144	RT-PCR, no data on Ct.
Yamahata	2020	Japan	Diamond Princess	3711 (2666 px, 1045 crew)	696	1 px	3711 (2666 px, 1045 crew)	696	18.8% of all px and crew	N/R	N/R	RT-PCR, no data on Ct.
Yeh	2021	USA	Diamond Princess	3711 px and crew	712	Unclear: 1 or 2	28 cases	712	N/A	N/R	N/R	RT-PCR, no data on Ct, GS (28 cases).
Abe	2022	Japan	Costa Atlantica	623 crew, 0 px	148 crew	1 crew	623 crew	147 crew	23.8% of all crew	N/R	N/R	RT-PCR positive, Ct < 40, GS.
Gravningen	2022	Norway	MS Roald Amundsen (Expedition 1 and 2)	Expedition 1: 210 px, 160 crew Expedition 2: 181 px, 160 crew	28 px. Expedition 1–3 px, Expedition 2–25 px. 42/167 crew	Unclear. Expedition1: 1 crew. Expedition 2: several crew	In study: 114 of 160 eligible crew. Total: 391 px, 167 crew	28/391 px, Expedition 1–3 px, Expedition 2–25 px. Crew- unclear	Px: 7.2% (1.4% in Expedition 1, 13.8% in Expedition 2). Crew: 25.2%	N/R	N/R	RT-PCR, Ct ≤ 37, GS.
Guagliardo	2020	USA	89 voyages on 70 ships; 16 ships had recurrent outbreaks.	145,460 px; 59,619 crew	1669 (px and crew) on the 89 voyages	N/R	Px data available for 57/89 voyages; crew data available for 52/89 voyages.	N/R	Attack rates on cruises, ranging from 13 to 62%.	N/R	N/R	RT-PCR, no data on Ct.
Ing	2020	Australia	N/R	223 (128 px, 95 crew)	128 (px and crew)	Unclear 1 to 6	217 (px and crew)	Unclear, up to 127 (px and crew)	59%	N/R	N/R	RT-PCR, no data on Ct.
Maeda	2021	Japan	Costa Atlantica	623 crew, 0 px	149 crew	Unclear. 1 crew	623	Unclear. Up to 148 confirmed cases, 107 probable cases	24% confirmed cases, 41% including probable cases	N/R	N/R	RT-PCR or LAMP, no data on Ct.
Moriarty	2020	USA	Grand Princess -B	3571 (2460 px; 1111 crew)	78 cases/469 cases with available results	Unclear	3571	Unclear	N/A	N/R	N/R	RT-PCR, no data on Ct.
Quigley—10 ships	2021	Australia	Diamond Princess, Ruby Princess, Ovation of the Seas, Voyager of the Seas, Celebrity Solstice, Artania, Costa Victoria, Silver Explorer, Greg Mortimer, Celebrity Eclipse	24862 px (including 2283 Australian px)	1908 px (including 957 Australian px)	N/R	10 142 Australian citizens	N/R	7.67%	N/R	N/R	RT-PCR positive, no data on Ct.
Quigley—36 ships	2021	Australia	36 ships	N/R	N/R	N/R	N/R	N/R	8.66%	N/R	N/R	RT-PCR positive, no data on Ct.
Sekizuka Cruise 1	2020	Japan	N/R (from Luxor to Awan)	N/R	N/R	N/R	3 px	3 px	N/A	N/R	N/R	RT-PCR positive, no data on Ct, GS.
Sekizuka Cruise 2	2020	Japan	N/R (from Awan to Luxor)	N/R	N/R	N/R	2 px	2 px	N/A	N/R	N/R	RT-PCR positive, no data on Ct, GS.

Abbreviations: px—passengers; Ct—cycle threshold; RT-PCR—real time reverse transcription–polymerase chain reaction; GS—genome sequencing.

**Table 2:** Environmental studies.

Study	Setting	Methods	Sample Source	Sample n/d	Live Cultures	Notes
Ahmed 2020	Cruise ship docked in Australia	Observational; sample collection occurred over a month after passenger disembarkation, with only crew onboard the ship on its last day berthed in Australia. Unconfirmed reports suggested as many as 24 infected persons may have been on board in the days prior to sample collection. Samples were transported on ice to the laboratory and stored at 4 ◦C and processed within 6–24 h after collection. To screen wastewater samples for SARS-CoV-2 RNA, the authors used two virus concentration methods (adsorption–extraction and Amicon^®^ Ultra-15 (30 kDa) Centrifugal Filter Device), five RT-qPCR assays (four targeting N gene and one targeting E gene), and one RT- ddPCR assay (targeting N gene). For the untreated wastewater collected from the cruise ship, all six replicate samples prepared using both virus concentration methods yielded a positive signal for SARS-CoV-2 RNA using the CDC N1 assay	Wastewater from cruise ship sanitation system; two wastewater grab samples (1 L) were collected from the influent and effluent of the membrane bioreactor of a cruise ship.	For the untreated wastewater collected from the cruise ship, all six replicate samples prepared using both virus concentration methods yielded a positive signal for SARS-CoV-2 RNA using the CDC N1 assay. The CDC N2 and NIID_2019-nCoV N assays detected SARS-CoV-2 RNA in four replicate samples. The E_Sarbeco assay appeared to be less analytically-sensitive (i.e., greater ALOD); only one of six replicates were RT-qPCR positive. The N_Sarbeco assay did not produce any amplification for these samples in two consecutive RT-qPCR runs. The CDC N1 and CDC N2 assays were consistently positive in replicate RT-qPCR reactions. The results showed positive SARS-CoV-2 signals, though concentrations were close to the limit of detection	N/A	For the adsorption–extraction method, the mean Cq value (Cq = 33.5) of the CDC N1 assay was much lower than the mean Cq value (Cq = 38) of CDC N2, E_Sarbeco, and NIID_2019-nCoV N. For ultrafiltration with the Amicon^®^ Ultra-15, the mean Cq value (Cq = 36.5) of the CDC N1 assay was slightly lower than the mean Cq value (Cq = 37.15) of CDC N2, E_Sarbeco, and NIID_2019-nCoV N assays. Among the replicate cruise ship untreated wastewater samples, four of six replicate samples were positive according to the CDC N1 RT-ddPCR assay. Of the five replicate cruise ship effluent wastewater samples (after treatment), Cq values ranged from 36.0 to 38.7. Greater concentrations were observed in the influent from the cruise ship in comparison with the effluent of the cruise ship. The frequency of SARS-CoV-2 RNA detection in treated cruise ship effluent wastewater was low in replicate RT-qPCR reactions compared with the cruise ship influent sample; this indicates that SARS-CoV-2 removal occurred in the wastewater treatment process.
Yamagishi 2020	Diamond Princess cruise ship on 22–23 February 2020	Environmental sampling, prior to disinfection of the vessel and while some passengers and crew members remained on board. Authors obtained specimens from cabins in which confirmed COVID-19 cases had stayed (case cabins), cabins with no confirmed case at any time (non-case cabins), and common areas. For sampling, they used polyester-flocked oropharyngeal specimen collection swabs moistened with viral transport medium (VTM). They swabbed areas (4 × 5 cm^2^) in 3 directions. Authors placed swabs into VTM and kept them frozen at −80°C until testing at National Institute of Infectious Diseases (NIID), Japan. Air samples (50 L/min for 20 min) were obtained from cabins by placing 2 air samplers (Airport MD8, Sartorius) in 7 random cabins on the bed and on the toilet seat. Collection was performed through a special gelatin filter (type 175, Sartorius; T1 phage capture rate, 99.99%; effective filtration cm^2^). After collection, the sample was put in the gelatin filter in the original package, checked, and stored at –80°C until testing at NIID (typically at least 14 days). Samples were tested by rRT-PCR.	For case cabins, authors randomly selected cabins in which confirmed symptomatic or asymptomatic COVID-19 cases had stayed. To understand the duration and survivability of SARS-CoV-2 on surfaces, the authors also selected case cabins according to the last date any person was in the cabin. Case cabins had been disinfected by 5% hydrogen peroxide spray prior to sampling (14–15 February 2020), including some of those that were sampled. To understand the contribution of airborne transmission, the authors selected non-case cabins next to a case cabin or at least 3 cabins away from a case cabin. To understand the contribution of wastewater, they also included non-case cabins located below case cabins. The authors swabbed diverse surfaces in cabins and common areas.	SARS-CoV-2 RNA was most often detected on the floor around the toilet in bathrooms (39%, 13/33; cycle quantification (Cq), 26.21–37.62) and bed pillows (34%, 11/32; Cq, 34.61–38.99). In case cabins occupied by symptomatic cases, SARS-CoV-2 RNA was detected in 15% (28/189) of samples tested, with Cq values ranging from 29.79 to 38.86. SARS-CoV-2 RNA was detected in 21% (28/131) of samples from case cabins with asymptomatic cases, with a range of Cq values from 26.21 to 38.99. All but 2 case cabins had 2 occupants before the room was vacated. The remaining 2 cabins had 1 and 3 occupants. The range of time between the last occupant vacating a case cabin and detection of SARS-CoV-2 RNA was 1–17 days, and rates of positivity decreased with time.	A second sampling of surfaces from part of the SARS-CoV-2 RNA-detected items was conducted on 27 February 2020 for viable virus isolation, with samples stored at 4 °C and transferred directly for laboratory isolation. The authors attempted viral isolation from some samples in which viral RNA had been detected by rRT-PCR and from the second round of sampling. Samples were mixed with Dulbecco’s modified Eagle medium. supplemented with standard concentrations of penicillin G, streptomycin, gentamicin, amphotericin B, and 5% fetal bovine serum. These were inoculated on confluent VeroE6/TMPRSS2 cells. Culture medium at 0 or 48 h post-infection was collected, diluted 10-fold in water, and boiled for 5 min. An rRT-PCR assay was performed to quantify the increased amount of coronavirus RNA with a MyGo Pro system (IT-IS Life Science). No viable virus could be isolated from the 58 samples with SARS-CoV-2 RNA detected by rRT-PCR or the 18 samples obtained in the second sampling.	The lowest Cq values were detected on samples taken 4 (Cq, 26.21) and 7 (Cq, 29.79) days after cabins were vacated, both obtained from the floor around the toilet. These findings suggest that environmental surfaces may have played a role in transmission of the virus. SARS-CoV-2 RNA was detected on multiple surfaces of case cabins, most often on bed pillows and the floor around the toilet in the bathroom, for up to 17 days, longer than previously reported. There was no difference in surface contamination between cabins of cases who were symptomatic and asymptomatic. It was evident that surface contamination occurred in rooms occupied by persons who were classified as being asymptomatic at the time they vacated their cabins. The high Cq values in most of the positive samples suggested low-level contamination of the environment after the COVID-19 cases vacated the cabins, potentially explaining why no virus was isolated.

Abbreviations: Ct—cycle threshold; Cq—quantification cycle; RT-PCR—real time reverse transcription-polymerase chain reaction.

**Table 3:** Quality assessment of included environmental studies.

Study	Study Type	Description of Methods with Sufficient Detail to Replicate	Sample Sources Clear	Analysis and Reporting Appropriate	Is Bias Dealt with	Applicability	Notes
Ahmed 2020	Observational	Yes	Yes	Yes	No	Yes	Cq values ranged from 36.0 to 38.7.
Yamagishi 2020	Observational	Yes	Yes	Yes	No	Yes	High Cq values in most of the positive samples. No viable virus could be isolated from the 58 samples with SARS-CoV-2 RNA detected by rRT-PCR or the 18 samples obtained in the second sampling.

Abbreviations: Ct—cycle threshold; Cq—quantification cycle; RT-PCR—real time reverse transcription–polymerase chain reaction.

**Table 4:** Quality assessment of included studies reporting on onboard transmission of SARS-CoV-2.

Study	Study Type	Clearly Defined Setting	Demographic Characteristics/Sampling Procedures Adequately Described	Follow-Up Strategy and Duration Sufficient for the Outcomes	The Transmission Outcomes Assessed Adequately	Main Threats to Validity Taken into Consideration?	Notes
Goldenfeld 2020	Observational	No	Unclear	No	Unclear	No	RT-PCR with data on Ct of 3 px. Ct considered positive—up to 40. Viral cultures in 1 px. Report on 6/15 repatriated Israeli citizens from Diamond Princess. Alternative exposures.
Hoshino 2021	Retrospective	No	Unclear	No	Unclear	No	Retrospective. All publicly available SARS-CoV-2 genome sequences with clinical information, as of 7 August, were retrieved from the Global Initiative on Sharing All Influenza Data (GISAID) database. RT-PCR positive, no data on Ct. The association between transmission dynamics and epidemiological factors could not be analyzed. Authors could not analyze the transmission dynamics in each subpopulation or between subpopulations. Potential sampling bias and sequencing errors due to next-generation sequencing techniques. It is difficult to obtain a complete and high-quality viral sequence from a sample with a low viral load.
Hoshiyama 2020	Retrospective	No	Unclear	No	Unclear	No	RT-PCR, no data on Ct. Bacterial cultures for co-infection.
Hung 2020	Prospective, observational	No	Yes	No	Yes	No	Follow-up for only 215 px from Hong Kong. Both parents and grandfather of case 7 tested positive for SARS-CoV-2 at the initial governmental screen in Japan.
Kakimoto 2020	Retrospective	Yes	Unclear	No	Unclear	No	RT-PCR, no data on Ct. Authors report findings from the initial phase of the cruise ship investigation into COVID-19 cases among crew members during 4–12 February 2020.
Moriarty 2020; Diamond Princess	Retrospective	No	Unclear	No	Unclear	No	RT-PCR, no data on Ct. Follow-up: 428 USA citizens out of 3711 px and crew. Alternative exposures (during repatriation).
Murata 2020	Observational	No	Yes	No	Yes	Yes	RT-PCR, viral cultures, GS. Authors followed 90 asymptomatic cases/3711 individuals. Timing of exposure among asymptomatic cases was not ascertained.
National Institute for Infectious Diseases Japan 2020	Retrospective	Yes	Unclear	Yes	Unclear	Yes	RT-PCR, no data on Ct. Some infections may have gone undetected. Asymptomatic infection early in the study period may have been underestimated if these asymptomatic case-patients cleared their viral loads before being tested. For some cases, symptom onset dates were obtained retrospectively. Greater than 9 persons who tested negative on the ship tested positive after being released.
Plucinski 2020	Retrospective, cross-sectional survey	Yes	Unclear	No	Unclear	No	RT-PCR, no data on Ct, GS (28 cases). The contribution that asymptomatic infected px played in the perpetuation of the outbreak could not be fully determined. Recall bias. Five percent of US citizens were never tested. US citizens made up 12% of the Diamond Princess population. Alternative exposures (during repatriation). Survey response rate of 52%.
Sekizuka 2020 Diamond Princess	Observational	No	Unclear	No	Unclear	No	RT-PCR with Cq, GS. No date of symptom onset. Follow-up for 24.11% of cases. Ct up to 40 considered positive. The Cq limit for successful GS determination was around 32.
Yamagishi 2020	Retrospective	Yes	Unclear	No	Unclear	No	RT-PCR, no data on Ct. Report on 490 individuals who were tested between 3 and 9 February. Testing strategy—only symptomatic cases and their contacts. Reporting bias.
Yamahata 2020	Observational, active case finding	Yes	Unclear	Yes	Unclear	Yes	RT-PCR, no data on Ct. Follow-up until 8 March.
Yeh 2021	Retrospective	No	Unclear	No	Unclear	No	RT-PCR, no data on Ct, GS for 28 cases from GISAID.
Walker 2021	Retrospective	Yes	Unclear	No	Unclear	No	Australian citizens made up 6% of the population on Diamond Princess. RT-PCR, no data on Ct. Alternative exposures (during repatriation). No asymptomatic testing was conducted in Australia.
Waltenburg 2021	Retrospective, longitudinal	No	Unclear	No	Unclear	No	Followed up only the US citizens from Diamond Princess (328 cases). RT-PCR with Ct < 40. Alternative exposures not excluded.
Abe 2022	Observational	No	No	Yes	Unclear	No	Diagnosis by RT-LAMP, with RT-PCR for positive samples (Ct < 40). GS for samples with Ct < 30 (complete sequencing for 94/148 samples).
Álvarez-León 2022	Observational	No	Unclear	No	Unclear	No	Unclear diagnostic test. Periodic antigen screening test. Pre-disembarkation screening was by antigen test. Alternative exposures before embarkation.
Maeda 2021	Retrospective	No	Unclear	Yes	Unclear	No	RT-PCR, with no data on Ct or LAMP. Unclear number of index cases. Possible underestimation of the number of laboratory-confirmed cases. Alternative exposures not excluded.
Gravningen 2022	Retrospective	Yes	Unclear	No	Unclear	No	Only 71% of crew members consented to participation and no px were included; data on social gatherings were not available. The symptom onset dates were obtained retrospectively for the early cases, which may have introduced selection and recall bias.
Guagliardo 2020	Retrospective	No	Unclear	No	Unclear	No	Px data available for 57/89 voyages; crew data available for 52/89 voyages. No data on index and secondary cases. RT-PCR with no data on Ct. Asymptomatic cases may have been missed. Voyage-level data extracted for each ship (duration, number of stops) may not be accurate, as authors relied on online resources for this information.
Ing 2020	Retrospective	Unclear	Unclear	Yes	Unclear	Yes	RT-PCR with no data on Ct. The number of index cases and secondary cases is not clear.
Moriarty 2020; Grand Princess	Retrospective	No	Unclear	No	Unclear	No	RT-PCR, no data on Ct. The number of index and secondary cases was unclear. Of 469 persons with available test results, 78 (16.6%) had positive test results for SARS-CoV-2. Authors assume that the index cases for the Voyage B were px and crew from the Voyage A. No alternative exposures excluded (e.g., infected px among the new px of Voyage B).
Quigley 2021—10 ships	Observational	No	Unclear	No	Unclear	No	RT-PCR, no data on Ct. Only symptomatic px were tested. No data on crew.
Quigley 2021—36 ships	Observational	Unclear	Unclear	Unclear	Unclear	No	RT-PCR, no data on Ct. A database of publicly available data was created for a total of 43 cruise ships with reported COVID-19 infected px during the study period. Data were sourced from news reports and cruise ship alerts. Due to missing passenger information, 7 ships were excluded from the analysis.
Sekizuka 2020 Cruise 1	Observational	No	Unclear	No	Unclear	No	RT-PCR, no data on Ct, GS. The study investigates only 3 px of a cruise ship.
Sekizuka 2020 Cruise 2	Observational	No	Unclear	No	Unclear	No	RT-PCR, no data on Ct, GS. The study investigated only 2 px from a cruise ship.

Abbreviations: Ct—cycle threshold; RT-PCR—real time reverse transcription-polymerase chain reaction. GS—genomic sequencing.

## Data Availability

All data included in the review are provided in the tables or the supplemental files.

## Supplementary Materials

The following supporting information can be downloaded at: https://www.mdpi.com/article/10.3390/tropicalmed7100290/s1, File S1; Updated Generic Protocol [53,54,55,56,57], File S2; Search strategy, File S3; Flow chart showing the process for inclusion of studies investigating cruise ships transmission, File S4; List of excluded studies, File S5; List of included studies, File S6; USA citizens repatriated from Diamond Princess, File S7: Timeline of events on Diamond Princess cruise ship [58,59], File S8; Characteristics of included studies, File S9; Genomic sequencing studies, File S10; Viral cultures.
