# Supplementary material for: Transmission of SARS-CoV-2 Associated with Cruise Ship Travel: A Systematic Review
Source: Trop Med Infect Dis. 2022 Oct 9;7(10):290. doi: 10.3390/tropicalmed7100290 (PMC9610645; doi:10.3390/tropicalmed7100290)
Supplement: Supplementary file 1 [file tropicalmed-07-00290-s001.zip › S10. Viral cultures.pdf]

**S10.** Viral cultures.

| Study | Participants | Methods | Results |
|---|---|---|---|
| Goldenfeld 2020 | 1 asymptomatic case with comorbidities | Nasal and throat swabs sampled on February 25, 2020 showed a notable cytopathic effect on Vero E6 cell culture. | Viral cultures positive 4 days after arrival.<br>Duration of RT-PCR positive test: 26 days. The viral load gradually decreased, but from day 17 in consecutive tests, varying viral loads were detected. The patient had two consecutive RT-PCR negative tests on days 26 and 27 of her hospitalization. |
| Murata 2021 | 7 asymptomatic carriers | Screening RT-PCR of nasopharyngeal or throat swabs. Samples with two or more positive PCR test results were subjected to viral culture. Nasopharyngeal swab specimens were transported in viral transport medium (BD universal viral transport system), and residual media were stored at 280°C after extraction of RNA for use in RT-PCR testing. These frozen media were thawed and processed for culture. The VeroE6/TMPRSS2 cells (Japanese Collection of Research Bioresources Cell Bank, number JCRB1819) were maintained in Dulbecco's modified Eagle's medium (DMEM) supplemented with 5% fetal bovine serum (FBS) and penicillin-streptomycin (Sigma-Aldrich). For isolation of SARS-CoV-2, cells were seeded on a 25-cm2 cell culture flask (Falcon). Next day, the thawed specimen medium (0.5 ml) was centrifuged at low speed, and the supernatant was | Of 166 nasopharyngeal samples collected from 39 asymptomatic carriers every 48 hours until two consecutive negative PCR test results were obtained, SARS-CoV-2 was successfully isolated from 9 PCR-positive samples which were obtained from 7 persons (18%; 7/39). Viable viruses were isolated predominantly within 7 days after the initial positive PCR test, except for one person who shed viable virus until day 15. The specimen in which CPE was observed 15 days following the initial positive PCR test was obtained from a 70-year-old female with a medical history of diabetes mellitus and hypertension, who had prolonged RT-PCR positivity for more than 21 days.<br>The median crossing point (Cp) value of RT-PCR of culture-positive samples was 24.6 (interquartile range [IQR], 20.4 to 25.8; range, 17.9 to 30.3). Cp values were significantly associated with isolation of viable virus (odds ratio, 0.496; 95% confidence interval [CI], 0.329 to 0.747; P value, 0.001). |

| | | | |
|---|---|---|---|
| | | mixed with 4.5 ml of isolation medium (DMEM supplemented with 2% FBS, penicillin-streptomycin [Sigma-Aldrich], gentamicin [Sigma-Aldrich], and amphotericin B [Sigma-Aldrich]). The maintenance medium in the flask was then removed, the cells were washed once with isolation medium, and the mixture (5 ml) was added to the flask, followed by incubation at 37°C. The authors checked whether the cells exhibited CPE, every day for 5 days. | |

*Abbreviations: RT-PCR - real time reverse transcription–polymerase chain reaction; CPE – cytopathic effect*
