# Supplementary material for: Transmission of SARS-CoV-2 Associated with Cruise Ship Travel: A Systematic Review
Source: Trop Med Infect Dis. 2022 Oct 9;7(10):290. doi: 10.3390/tropicalmed7100290 (PMC9610645; doi:10.3390/tropicalmed7100290)
Supplement: Supplementary file 1 [file tropicalmed-07-00290-s001.zip › S2. Search strategy. Cruise ships.pdf]

Supplementary Material S2: Search Strategy

We searched four main databases:

- **WHO Covid-19 Database** (https://search.bvsalud.org/global-literature-on-novel-coronavirus-2019-ncov/)
  The global literature cited in the WHO COVID-19 database is updated daily (Monday through Friday) from searches of bibliographic databases, hand searching, and the addition of other expert-referred scientific articles.
- **LitCovid** (https://www.ncbi.nlm.nih.gov/research/coronavirus/)
  A curated literature hub for tracking up-to-date scientific information about the 2019 novel Coronavirus. It is a comprehensive resource on the subject, providing a central access to relevant articles in PubMed.
- **medRxiv** (https://www.medrxiv.org/)
  A free online archive and distribution server for complete but unpublished manuscripts (preprints) in the medical, clinical, and related health sciences.
- **Google Scholar** (https://scholar.google.com/)
  Provides a broad search for scholarly literature across many disciplines and sources: articles, theses, books, abstracts and court opinions, from academic publishers, professional societies, online repositories, universities and other web sites.

Searches were initially undertaken on a monthly basis, as per the Open Evidence Review process. However, from September 2020 due to the volume of new studies, they moved to searching every fortnight until 26 May 2022. For databases which do not support date granularity, date of publication was approximated. All retrieved articles are entered into a google sheet that is then used to filter and check articles for inclusion.

WHO Covid-19 Database, LitCovid are specific Covid-19 databases so there was no requirement to add a search string to identify Covid-19 articles. For medRxiv and Google Scholar we used the terms (*coronavirus* OR *covid-19* OR *SARS-CoV-2*) AND (*cruise* OR *cruiseship* OR *ship* OR *liner*) AND *transmission*. For WHO Covid-19 Database and LitCovid we used the terms (*cruise* OR *cruiseship* OR *ship* OR *liner*) AND *transmission*.

Additional techniques were used to identify relevant topics:

- For articles that looked particularly relevant, citation tracking was undertaken.

The search strategy is subject to continual review and updated and is maintained by Brassey J.
