# Supplementary material for: Transmission of SARS-CoV-2 Associated with Cruise Ship Travel: A Systematic Review
Source: Trop Med Infect Dis. 2022 Oct 9;7(10):290. doi: 10.3390/tropicalmed7100290 (PMC9610645; doi:10.3390/tropicalmed7100290)
Supplement: Supplementary file 1 [file tropicalmed-07-00290-s001.zip › S3. Flow chart showing the process for inclusion of studies investigating cruise ships transmission.pptx]

## Slide 1
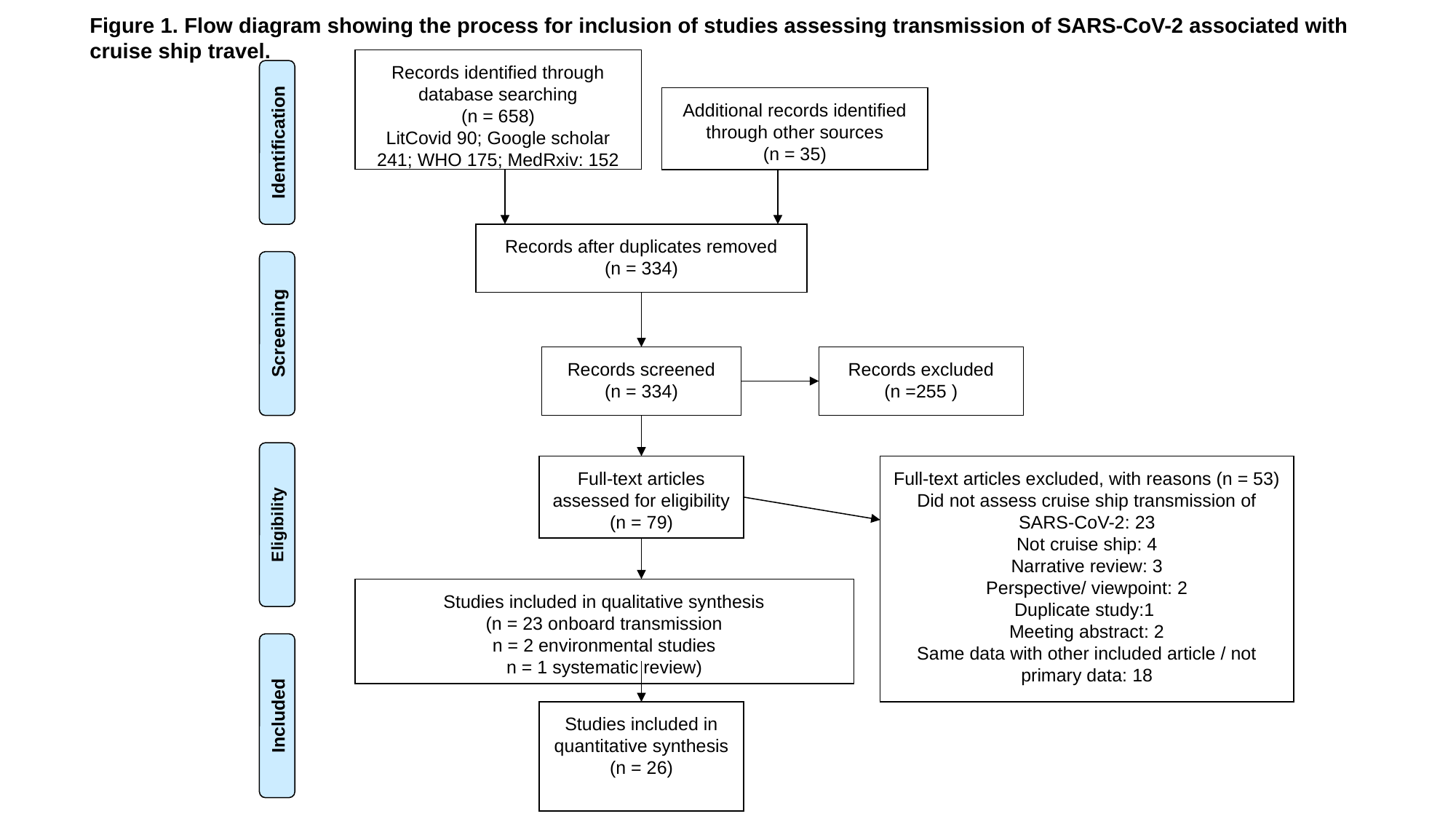

Figure 1. Flow diagram showing the process for inclusion of studies assessing transmission of SARS-CoV-2 associated with cruise ship travel.
Records identified through database searching
(n = 658)
LitCovid 90; Google scholar 241; WHO 175; MedRxiv: 152
Additional records identified through other sources
(n = 35)
Identification
Records after duplicates removed
(n = 334)
Screening
Records screened
(n = 334)
Records excluded
(n =255 )
Full-text articles excluded, with reasons (n = 53)
Did not assess cruise ship transmission of SARS-CoV-2: 23
Not cruise ship: 4
Narrative review: 3
Perspective/ viewpoint: 2
Duplicate study:1
Meeting abstract: 2
Same data with other included article / not primary data: 18
Full-text articles assessed for eligibility
(n = 79)
Eligibility
Studies included in qualitative synthesis
(n = 23 onboard transmission
n = 2 environmental studies
n = 1 systematic review)
Included
Studies included in quantitative synthesis (n = 26)
