# Supplementary material for: Transmission of SARS-CoV-2 Associated with Cruise Ship Travel: A Systematic Review
Source: Trop Med Infect Dis. 2022 Oct 9;7(10):290. doi: 10.3390/tropicalmed7100290 (PMC9610645; doi:10.3390/tropicalmed7100290)
Supplement: Supplementary file 1 [file tropicalmed-07-00290-s001.zip › S4. List of excluded studies - cruise ships.pdf]

Supplementary Material S4

List of Excluded studies

1. Almilaji, O. Air recirculation role in the spread of COVID-19 onboard the Diamond Princess cruise ship during a quarantine period. Aerosol Air Qual. Res 2021; 21, 200495. https://doi.org/10.4209/aaqr.200495 - data from National Institute of Infectious Disease (NIID) 20 Feb 2020
2. Anan H, Kondo H, Takeuchi I, Nakamori T, Ikeda Y, Akasaka O, Koido Y. Medical transport for 769 COVID-19 patients on a cruise ship by Japan Disaster Medical Assistance Team. Disaster Med Public Health Prep. 2020; 14(6): e47-e50. doi: 10.1017/dmp.2020.187. – not a transmission study; report on medical transportation of passengers
3. Arashiro T, Nakamura S, Asami T, Mikuni H, Fujiwara E, Sakamoto S, Miura R, Shionoya Y, Honda R, Furukawa K, Nakamura A, Saito H. SARS-CoV-2 and Legionella co-infection in a person returning from a Nile cruise. J Travel Med. 2020;27(3): taaa053. doi: 10.1093/jtm/taaa053. Case report; not assessing transmission
4. Arashiro T, Furukawa K, Nakamura A. COVID-19 in 2 persons with mild upper respiratory tract symptoms on a cruise ship, Japan. Emerg Infect Dis. 2020;26(6): 1345-1348. doi: 10.3201/eid2606.200452. Two case reports
5. Boogaerts HLF, Smits P, Hans G, Bouly L, Coeck E, Vandamme S, Jansens H, Goossens H, Matheeussen V. Laboratory analysis of two Delta SARS-CoV-2 variant outbreaks in the Port of Antwerp. Acta Clin Belg. 2021:1-8. doi: 10.1080/17843286.2021.2010966. – not cruise ship study
6. Chowell G, Dahal S, Bono R, Mizumoto K. Harnessing testing strategies and public health measures to avert COVID-19 outbreaks during ocean cruises. Sci Rep. 2021;11(1):15482. doi: 10.1038/s41598-021-95032-4. – modelling study with data from Diamond Princess
7. E Chrysikou, E Hernandez Garcia, E Savvopoulou, J Haldane, H Satoh, Environment related practices for emergency response to infectious disease outbreak on cruise ships, European Journal of Public Health, Volume 31, Issue Supplement_3, ckab165.059, https://doi.org/10.1093/eurpub/ckab165.059 - meeting abstract
8. Draper AD, Dempsey KE, Boyd RH, Childs EM, Black HM, Francis LA, Markey PG, Krause VL. The first 2 months of COVID-19 contact tracing in the Northern Territory of Australia. Commun Dis

Intell (2018). 2020;44. doi: 10.33321/cdi.2020.44.53. PMID: 32615916. – study on contact tracing (close contacts from a cruise ship)

9. Emery JC, Russell TW, Liu Y, Hellewell J, Pearson CA; CMMID COVID-19 Working Group, Knight GM, Eggo RM, Kucharski AJ, Funk S, Flasche S, Houben RM. The contribution of asymptomatic SARS-CoV-2 infections to transmission on the Diamond Princess cruise ship. Elife. 2020; 9:e58699. doi: 10.7554/eLife.58699. – modelling with data from Mizumoto 2020, Nishiura 2020, NIID 2020
10. Gupta A, Kunte R, Goyal N, Ray S, Singh K. A comparative analysis of control measures on-board ship against COVID-19 and similar novel viral respiratory disease outbreak: Quarantine ship or disembark suspects?. Med J Armed Forces India. 2020; 10.1016/j.mjafi.2020.06.003. doi:10.1016/j.mjafi.2020.06.003 – review, with data from the Japanese Ministry of Health, Labour and Welfare and Moriartry 2020 on Diamond Princess
11. Gupta H, Patwa AK, Kumar S, Gupta A. Transmission data of Covid 19- Lessons of diamond princess cruise ship event. J Family Med Prim Care. 2021;10(12):4611-4612. doi: 10.4103/jfmpc.jfmpc_1337_21. – letter to editor on Diamond Princess; no primary data
12. Harvey RR, Nett RJ, McNamara K, McClung RP, Pieracci EG, Mayer O, Labar KA, Xu K, Facey J, Honein MA. Influenza-Like Illness Among Personnel Responding to U.S. Quarantine of Cruise Ship Passengers Exposed to SARS-CoV-2. J Occup Environ Med. 2022;64(1):58-63. doi: 10.1097/JOM.0000000000002335. – not cruise ships
13. Hasan T, Lim HL, Case J, Hueston L, Bag S, Dwyer DE, O'Sullivan MVN. The utility of SARS-CoV-2-specific serology in COVID-19 diagnosis. Aust N Z J Public Health. 2021;45(6):616-621. doi: 10.1111/1753-6405.13155. – not cruise ships
14. Hogarth FG, Nye R, Pingault N, Crouch S, Coffey C, Smith K, et al. COVID-19 outbreaks in Australia during a period of high epidemic control, 2020. MedRxiv 2022. doi:10.1101/2022.02.07.22270575. – outbreaks in Australia, not a cruise ship transmission study
15. Holland J, Mazzarol T, Soutar GN, Tapsall S, Elliott WA. Cruise passengers' risk reduction strategies in the wake of COVID-19. Asia Pacific Journal of Tourism Research 2021;26:1189–206. doi:10.1080/10941665.2021.1962376. – consumer survey, not cruise ship transmission study
16. Inui S, Fujikawa A, Jitsu M, et al. Chest CT findings in cases from the cruise ship Diamond Princess with Coronavirus Disease (COVID-19) [published correction appears in Radiol Cardiothorac Imaging. 2020 Apr 07;2(2):e204002]. Radiol Cardiothorac Imaging.

2020;2(2):e200110. doi:10.1148/ryct.2020200110. – study on the chest CT of patients from Diamond Princess

17. Jenness SM, Willebrand KS, Malik AA, Lopman BA, Omer SB. Dynamic network strategies for SARS-CoV-2 control on a cruise ship. Epidemics. 2021;37:100488. doi: 10.1016/j.epidem.2021.100488. – modelling study with data from Diamond Princess; no primary data
18. Jimi H, Hashimoto G. Challenges of COVID-19 outbreak on the cruise ship Diamond Princess docked at Yokohama, Japan: a real-world story. Glob Health Med. 2020;2(2):63-65. doi:10.35772/ghm.2020.01038 - Diamond Princess - mainly describes the events during the quarantine period from 5 to 23 February 2020, when the last group of the quarantined passengers left the ship
19. Johnston FH, Anderson T, Harlock M, Castree N, Parry L, Marfori T, McPherson M, Veitch M, Smith KJ, Stephens N. Lessons learnt from the first large outbreak of COVID-19 in health-care settings in Tasmania, Australia. Western Pac Surveill Response J. 2021;12(4):1-7. doi: 10.5365/wpsar.2021.12.4.884.– not cruise ship transmission
20. Jorden MA, Rudman SL, et al. Evidence for limited early spread of COVID-19 within the United States, January–February 2020. MMWR Morb Mortal Wkly Rep 2020;69:680–684. DOI: http://dx.doi.org/10.15585/mmwr.mm6922e1 - Surveillance report
21. Kato H, Shimizu H, Shibue Y, Hosoda T, Iwabuchi K, Nagamine K, Saito H, Sawada R, Oishi T, Tsukiji J, Fujita H, Furuya R, Masuda M, Akasaka O, Ikeda Y, Sakamoto M, Sakai K, Uchiyama M, Watanabe H, Yamaguchi N, Higa R, Sasaki A, Tanaka K, Toyoda Y, Hamanaka S, Miyazawa N, Shimizu A, Fukase F, Iwai S, Komase Y, Kawasaki T, Nagata I, Nakayama Y, Takei T, Kimura K, Kunisaki R, Kudo M, Takeuchi I, Nakajima H. Clinical course of 2019 novel coronavirus disease (COVID-19) in individuals present during the outbreak on the Diamond Princess cruise ship. J Infect Chemother. 2020;26(8):865-869. doi: 10.1016/j.jiac.2020.05.005. – Clinical findings of 70 hospitalized cases from Diamond Princess
22. Kobayashi T, Yoshii K, Linton NM, Suzuki M, Nishiura H. Age dependence of the natural history of infection with severe acute respiratory syndrome coronavirus 2 (SARS-CoV-2): an analysis of Diamond Princess data. Int J Infect Dis. 2022;115:109-115. doi: 10.1016/j.ijid.2021.12.319. – study with data from other articles (e.g., Expert Taskforce for the COVID-19 Cruise Ship Outbreak.); no primary data

23. La Scola B, Lavrard P, Fournier PE, Colson P, Lacoste A, Raoult D. SARS-CoV-2 variant from India to Marseille: The still active role of ports in the introduction of epidemics. Travel Med Infect Dis. 2021;42:102085. doi: 10.1016/j.tmaid.2021.102085. – not cruise ship transmission study
24. Leung WS, Chan JMC, Chik TSH, Lau DPL, Choi CYC, Lau AWT, Tsang OTY. Presumed COVID-19 index case on diamond princess cruise ship and evacuees to Hong Kong. J Travel Med. 2020;27(5): taaa073. doi: 10.1093/jtm/taaa073. – Letter to editor summarizing the diagnosis and management of the first passenger tested positive for SARS-CoV-2 after disembarking from the cruise ship and the epidemiological features of the Hong Kong evacuees diagnosed COVID-19 after returning to Hong Kong
25. Linton NM, Akhmetzhanov AR, Nishiura H. Correlation between times to SARS-CoV-2 symptom onset and secondary transmission undermines epidemic control efforts. MedRxiv 2021. doi:10.1101/2021.08.29.21262512.- not cruise ships
26. Liu, S-F, Kuo, N-Y, Kuo, H-C. Three Taiwan's domestic family cluster infections of coronavirus disease 2019. J Med Virol. 2020; 92: 2011– 2018. https://doi.org/10.1002/jmv.25949 - not cruise ships
27. Liu F, Li X, Zhu G. Using the contact network model and Metropolis-Hastings sampling to reconstruct the COVID-19 spread on the "Diamond Princess". Sci Bull (Beijing). 2020;65(15):1297-1305. doi:10.1016/j.scib.2020.04.043 – modelling with data from Diamond Princess
28. Mizumoto K, Chowell G. Transmission potential of the novel coronavirus (COVID-19) onboard the diamond Princess Cruises Ship, 2020. Infect Dis Model. 2020 Feb 29;5:264-270. doi: 10.1016/j.idm.2020.02.003. PMID: 32190785; PMCID: PMC7068636. – Modelling with data from Diamond Princess
29. Mizumoto K, Kagaya K, Zarebski A, Chowell G. Estimating the asymptomatic proportion of coronavirus disease 2019 (COVID-19) cases on board the Diamond Princess cruise ship, Yokohama, Japan, 2020. Euro Surveill. 2020;25(10):2000180. doi: 10.2807/1560-7917.ES.2020.25.10.2000180. Erratum in: Euro Surveill. 2020 Jun;25(22): PMID: 32183930; PMCID: PMC7078829. – Modelling with data from Diamond Princess
30. Miyamae Y, Hayashi T, Yonezawa H, Fujihara J, Matsumoto Y, Ito T, Tsubota T, Ishii K. Duration of viral shedding in asymptomatic or mild cases of novel coronavirus disease 2019 (COVID-19) from a cruise ship: A single-hospital experience in Tokyo, Japan. Int J Infect Dis. 2020;97:293-295. doi: 10.1016/j.ijid.2020.06.020. – 23 Cases from Diamond Princess

31. Morciglio A, Zhang B, Chowell G, Hyman JM, Jiang Y. Mask-Ematics: Modeling the Effects of Masks in COVID-19 Transmission in High-Risk Environments. Epidemiologia 2021;2:207–26. doi:10.3390/epidemiologia2020016. – modelling study with data from Diamond Princess; not primary data
32. Naik AQ, Zafar T, Shrivastava VK. The perspective of coronavirus disease outbreak: epidemiology, transmission, and possible treatment. Vector Borne Zoonotic Dis. 2021; 21(2):78-85. doi: 10.1089/vbz.2020.2678. – Perspective
33. National Institute of Japan. Field Briefing: Diamond Princess COVID-19 Cases, 20 Feb Update. Available at: https://www.niid.go.jp/niid/en/2019ncov-e/9417-covid-dp-fe-02.html - a more recent report has been published: Epidemiology of COVID-19 Outbreak on Cruise Ship Quarantined at Yokohama, Japan, February 2020. Emerg Infect Dis. 2020;26(11):2591-2597. https://doi.org/10.3201/eid2611.201165
34. Nakazawa E, Ino H, Akabayashi A. Chronology of COVID-19 cases on the Diamond Princess cruise ship and ethical considerations: a report from Japan. Disaster Med Public Health Prep. 2020;14(4):506-513. doi:10.1017/dmp.2020.50. – Only chronology of SARS-CoV-2 infection from Diamond Princess
35. Nishiura H, Kobayashi T, Yang Y, Hayashi K, Miyama T, Kinoshita R, Linton NM, Jung SM, Yuan B, Suzuki A, Akhmetzhanov AR. The rate of underascertainment of novel coronavirus (2019-nCoV) infection: estimation using Japanese passengers data on evacuation flights. J Clin Med. 2020; 9(2):419. doi: 10.3390/jcm9020419. – Not cruise ships
36. Palafox NA, Best BR, Hixon A, Alik WC. Viewpoint: Pacific Voyages - ships - Pacific communities: a framework for COVID-19 prevention and control. Hawaii J Health Soc Welf. 2020;79(6 Suppl 2):120-123. – viewpoint
37. Palafox NA, Garcia MT, Chutaro E, Alailima C, Hixon AL, Silk I, Best B, Alik WC, Tufa AJ, Cash HL. COVID-19 Containment Ship Model: A Case Study for Pacific Island Response. Hawaii J Health Soc Welf. 2021;80(9 Suppl 1):102-109. – containment ship models, not a transmission study
38. Pung R, Firth JA, Spurgin LG; Singapore CruiseSafe working group; CMMID COVID-19 working group, Lee VJ, Kucharski AJ. Using high-resolution contact networks to evaluate SARS-CoV-2 transmission and control in large-scale multi-day events. Nat Commun. 2022 ;13(1):1956. doi: 10.1038/s41467-022-29522-y. – not cruise ship transmission study
39. Rocklöv J, Sjödin H, Wilder-Smith A. COVID-19 outbreak on the Diamond Princess cruise ship: estimating the epidemic potential and effectiveness of public health countermeasures. J Travel

Med. 2020; 27(3):taaa030. doi: 10.1093/jtm/taaa030. Diamond Princess – data from The Princess Cruises' official website and National Institute of Infectious Diseases, Japan, official website.

40. Sahu A, Naqvi WM. Floating countries and corona pandemic: Impact of covid-19 on stranded cruise ships. Int. J. Res. Pharm. Sci. 2020; SI 1(11): 219-223, 20200311. – Narrative review
41. Salducci M, La Torre G. COVID-19 emergency in the cruise's ship: a case report of conjunctivitis. Clin Ter. 2020; 171(3): e189-e191. doi: 10.7417/CT.2020.2212. – Case report from Diamond Princess
42. Sando E, Morimoto K, Narukawa S, Nakata K. COVID-19 outbreak on the Costa Atlantica cruise ship: use of a remote health monitoring system. J Travel Med. 2021 Feb 23;28(2):taaa163. doi: 10.1093/jtm/taaa163. - Letter to Editor reporting the use of a remote health monitoring system. The Costa Atlantica outbreak is presented in more detail by another included paper, with focus on cruise ship transmission (Maeda H, Sando E, Toizumi M, et al. Epidemiology of Coronavirus Disease Outbreak among Crewmembers on Cruise Ship, Nagasaki City, Japan, April 2020. Emerg Infect Dis. 2021; 27(9):2251-2260. doi: 10.3201/eid2709.204596.)
43. Schuchat A. Public Health response to the initiation and spread of pandemic COVID-19 in the United States, February 24–April 21, 2020. MMWR Morb Mortal Wkly Rep 2020; 69:551–556. DOI: http://dx.doi.org/10.15585/mmwr.mm6918e2. – Public Health report; no data can be extracted
44. Motoi Suzuki, Hajime Kamiya, Kiyoko Okamoto, Takuya Yamagishi, Kensaku Kakimoto, Makoto Takeda, Syutoku Matsuyama, Kazuya Shirato, Naganori Nao, Hideki Hasegawa, Tsutomu Kageyama, Ikuyo Takayama, Shinji Saito, Takaji Wakita, Makoto Ohnishi, Koji Wada, Retsu Fujita, Yoshiaki Gu, Nobuaki Matsunaga, Mikiyo Sakaguchi, R.N. Taichi Tajima, Norio Ohmagari, Saho Takaya, Hiroki Saito, Keiji Okinaka, Mathew Griffith, Amy Elizabeth Parry, Mateusz M Plucinski, Brenda Barnetson, James Leonard. Environmental sampling for severe acute respiratory syndrome coronavirus 2 (SARS-CoV-2) during a coronavirus disease (COVID-19) outbreak aboard a commercial cruise ship. medRxiv 2020.05.02.20088567; doi: https://doi.org/10.1101/2020.05.02.20088567. – Preprint of Yamagishi 2020-2
45. Pengcheng Xu, Hua Qian, Te Miao, Hui-Ling Yen, Hongwei Tan, Min Kang, Benjamin J. Cowling, Yuguo Li. Transmission routes of Covid-19 virus in the Diamond Princess cruise ship. medRxiv 2020.04.09.20059113; doi: https://doi.org/10.1101/2020.04.09.20059113 Modelling study, with data from the Ministry of Health, Labour and Welfare, Japan. Preprint of Xu 2021

46. Tabata S, Imai K, Kawano S, Ikeda M, Kodama T, Miyoshi K, Obinata H, Mimura S, Kodera T, Kitagaki M, Sato M, Suzuki S, Ito T, Uwabe Y, Tamura K. Clinical characteristics of COVID-19 in 104 people with SARS-CoV-2 infection on the Diamond Princess cruise ship: a retrospective analysis. Lancet Infect Dis. 2020 Sep;20(9):1043-1050. doi: 10.1016/S1473-3099(20)30482-5. A summary of the clinical characteristics of 104 participants with laboratory-detected SARS-CoV-2 infection from Diamond Princess, who were treated at Self-Defense Forces Central Hospital, Japan, from Feb 11 to Feb 25, 2020. No on-board transmission data.
47. Wang Z, Meng C, Yao M, Claramunt C. Modelling the Risk of Imported COVID-19 Infections at Maritime Ports Based on the Mobility of International-Going Ships. ISPRS International Journal of Geo-Information. 2022; 11(1):60. https://doi.org/10.3390/ijgi11010060 - modelling study, not a cruise ship transmission study
48. Willebrand K, Pischel L, Malik AA, Jenness S, Omer S. A Systematic Review of COVID-19 Transmission Dynamics and Clinical Response on Cruise Ships Globally Between January and October 2020. Open forum infectious diseases ; 8(Suppl 1):S294-S295, 2021. – meeting abstract
49. Willebrand KS, Pischel L, Malik AA, Jenness SM, Omer SB. A review of COVID-19 transmission dynamics and clinical outcomes on cruise ships worldwide, January to October 2020. Euro Surveill. 2022; 27(1):2002113. doi: 10.2807/1560-7917.ES.2022.27.1.2002113. - The authors performed a systematic search in PubMed, and found additional records through other sources; the database compiled by the Miami Herald of news reports and the list of cruise ships reporting COVID-19 outbreaks provided by the US CDC were used as foundational material. All sources were checked and expanded upon via Google News searches of the cruise ship name and keywords. The present review is not a systematic review as the authors searched only one literature database (PubMed) and did not assess the risk of bias. Furthermore, the reports from news websites are not reliable sources for research. For example, authors stated: "when the number of guests or crew on board was not explicitly stated, the number of passengers was estimated from Securities and Exchange Commission (SEC) reports", "Total COVID-19 cases may not equal the sum of crew and guest cases because some reports did not identify cases as crew members or guests.", "Numbers are approximate; when number of guests was not known, the maximum occupancy of guests was used."
50. Xu P, Jia W, Qian H, et al. Lack of cross-transmission of SARS-CoV-2 between passenger's cabins on the Diamond Princess cruise ship. Build Environ. 2021;198:107839.

doi:10.1016/j.buildenv.2021.107839. Modelling study, with data from the Ministry of Health, Labour and Welfare, Japan

51. Yang W, Li C, Wang F, Dou K, Cheng Y, Ni B, Hou X. [Retrospective analysis of the on-site treatment of the coronavirus disease 2019 epidemic on the Costa Crociere cruise]. Zhonghua Wei Zhong Bing Ji Jiu Yi Xue. 2020 Jun;32(6):750-753. Chinese. doi: 10.3760/cma.j.cn121430-20200223-00162. Not a SARS-CoV-2 transmission study
52. Yoshimura Y, Sasaki H, Horiuchi H, Miyata N, Tachikawa N. Clinical characteristics of the coronavirus disease 2019 (COVID-19) outbreak on a cruise ship. J Infect Chemother. 2020;26(11):1177-1180. doi:10.1016/j.jiac.2020.06.010 - retrospective observational study of the COVID- 19 cases from Diamond Princess who were admitted to the Yokohama Municipal Citizen's (17 cases)
53. Zhang S, Diao M, Yu W, Pei L, Lin Z, Chen D. Estimation of the reproductive number of novel coronavirus (COVID-19) and the probable outbreak size on the Diamond Princess cruise ship: A data-driven analysis. Int J Infect Dis. 2020; 93:201-204. doi: 10.1016/j.ijid.2020.02.033. - Modelling study with data from the Ministry of Health, Labour and Welfare of Japan, 2020)
