# Supplementary material for: Transmission of SARS-CoV-2 Associated with Cruise Ship Travel: A Systematic Review
Source: Trop Med Infect Dis. 2022 Oct 9;7(10):290. doi: 10.3390/tropicalmed7100290 (PMC9610645; doi:10.3390/tropicalmed7100290)
Supplement: Supplementary file 1 [file tropicalmed-07-00290-s001.zip › S5. List of included studies - cruise ships.pdf]

Supplementary Material S5 - List of included studies

On-board transmission

1. Abe H, Ushijima Y, Amano M, Sakurai Y, Yoshikawa R, Kinoshita T, Kurosaki Y, Yanagihara K, Izumikawa K, Morita K, Kohno S, Yasuda J. Unique Evolution of SARS-CoV-2 in the Second Large Cruise Ship Cluster in Japan. Microorganisms. 2022;10(1):99. doi: 10.3390/microorganisms10010099.
2. Álvarez-León EE, Fernández-Nakoura DM, López-Villarrubia E, Alemán-Sánchez JJ. Real-world experience on cruise ships in the Canary Islands highlights that safe travel is possible. J Travel Med. 2022;29(3):taac001. doi: 10.1093/jtm/taac001.
3. Goldenfeld M, Nir-Paz R, Segal G, Bar-On E, Mendelson E, Mandelboim M, Wolf DG, Marom EM, Israely T, Achdout H, Rahav G, Hanage WP, Regev-Yochay G. Characteristics of clinically asymptomatic patients with SARS-CoV-2 infections, Case series. Prehosp Disaster Med. 2021;36(1):125-128. doi: 10.1017/S1049023X20001466.
4. Gravningen K, Henriksen S, Hungnes O, Svendsen K, MacDonald E, Schirmer H, Stene-Johansen K, Simonsen GS, Kacelnik O, Elstrøm P, Bragstad K, Rinaldo CH. Risk factors, immune response and whole-genome sequencing of SARS-CoV-2 in a cruise ship outbreak in Norway. Int J Infect Dis. 2022;118:10-20. doi: 10.1016/j.ijid.2022.02.025.
5. Guagliardo SAJ, Prasad PV, Rodriguez A, Fukunaga R, Novak RT, Ahart L, Reynolds J, Griffin I, Wiegand R, Quilter LAS, Morrison S, Jenkins K, Wall HK, Treffiletti A, White SB, Regan J, Tardivel K, Freeland A, Brown C, Wolford H, Johansson MA, Cetron MS, Slayton RB, Friedman CR. Cruise ship travel in the era of COVID-19: A summary of outbreaks and a model of public health interventions. Clin Infect Dis. 2021; ciab433. doi: 10.1093/cid/ciab433.
6. Hoshino K, Maeshiro T, Nishida N, Sugiyama M, Fujita J, Gojobori T, Mizokami M. Transmission dynamics of SARS-CoV-2 on the Diamond Princess uncovered using viral genome sequence analysis. Gene. 2021; 779:145496. doi: 10.1016/j.gene.2021.145496.
7. Hoshiyama T, Wada T, Nihonyanagi S, Kameda R, Yamaoka-Tojo M, Fukuda M, Ako J, Yamaoka K, Takayama Y. Clinical and microbiological features of asymptomatic SARS-CoV-2 infection and mild COVID-19 in seven crewmembers of a cruise ship. Intern Med. 2020; 59(24):3135-3140. doi: 10.2169/internalmedicine.5601-20.
8. Hung IF, Cheng VC, Li X, Tam AR, Hung DL, Chiu KH, Yip CC, Cai JP, Ho DT, Wong SC, Leung SS, Chu MY, Tang MO, Chen JH, Poon RW, Fung AY, Zhang RR, Yan EY, Chen LL, Choi CY, Leung KH, Chung TW, Lam SH, Lam TP, Chan JF, Chan KH, Wu TC, Ho PL, Chan JW, Lau CS, To KK, Yuen KY. SARS-CoV-2 shedding and seroconversion among passengers quarantined after disembarking a cruise ship: a case series. Lancet Infect Dis. 2020; 20(9):1051-1060. doi: 10.1016/S1473-3099(20)30364-9.
9. Ing AJ, Cocks C, Green JP. COVID-19: in the footsteps of Ernest Shackleton. Thorax. 2020; 75(8):693-694. doi: 10.1136/thoraxjnl-2020-215091.
10. Kakimoto K, Kamiya H, Yamagishi T, Matsui T, Suzuki M, Wakita T. Initial investigation of transmission of COVID-19 among crew members during quarantine of a cruisesShip - Yokohama, Japan, February 2020. MMWR Morb Mortal Wkly Rep. 2020 Mar 20;69(11):312-313. doi: 10.15585/mmwr.mm6911e2. Erratum in: MMWR Morb Mortal Wkly Rep. 2020; 69(13):389.
11. Maeda H, Sando E, Toizumi M, Arima Y, Shimada T, Tanaka T, Tashiro M, Fujita A, Yanagihara K, Takayama H, Yasuda I, Kawachi N, Kohayagawa Y, Hasegawa M, Motomura K, Fujita R, Nakata K, Yasuda J, Morita K, Kohno S, Izumikawa K, Suzuki M, Morimoto K. Epidemiology of Coronavirus Disease Outbreak among Crewmembers on Cruise Ship, Nagasaki City, Japan, April 2020. Emerg Infect Dis. 2021;27(9):2251-2260. doi: 10.3201/eid2709.204596.

12. Moriarty LF, Plucinski MM, Marston BJ, Kurbatova EV, Knust B, Murray EL, Pesik N, Rose D, Fitter D, Kobayashi M, Toda M, Cantey PT, Scheuer T, Halsey ES, Cohen NJ, Stockman L, Wadford DA, Medley AM, Green G, Regan JJ, Tardivel K, White S, Brown C, Morales C, Yen C, Wittry B, Freeland A, Naramore S, Novak RT, Daigle D, Weinberg M, Acosta A, Herzig C, Kapella BK, Jacobson KR, Lamba K, Ishizumi A, Sarisky J, Svendsen E, Blocher T, Wu C, Charles J, Wagner R, Stewart A, Mead PS, Kurylo E, Campbell S, Murray R, Weidle P, Cetron M, Friedman CR; CDC Cruise Ship Response Team; California Department of Public Health COVID-19 Team; Solano County COVID-19 Team. Public Health Responses to COVID-19 Outbreaks on Cruise Ships - Worldwide, February-March 2020. MMWR Morb Mortal Wkly Rep. 2020; 69(12):347-352. doi: 10.15585/mmwr.mm6912e3.
13. Murata T, Sakurai A, Suzuki M, Komoto S, Ide T, Ishihara T, Doi Y. Shedding of viable virus in asymptomatic SARS-CoV-2 carriers. mSphere. 2021; 6(3):e00019-21.
14. Expert Taskforce for the COVID-19 Cruise Ship Outbreak. Epidemiology of COVID-19 outbreak on cruise ship quarantined at Yokohama, Japan, February 2020. Emerg Infect Dis. 2020; 26(11):2591-2597. doi: 10.3201/eid2611.201165.
15. Plucinski MM, Wallace M, Uehara A, Kurbatova EV, Tobolowsky FA, Schneider ZD, Ishizumi A, Bozio CH, Kobayashi M, Toda M, Stewart A, Wagner RL, Moriarty LF, Murray R, Queen K, Tao Y, Paden C, Mauldin MR, Zhang J, Li Y, Elkins CA, Lu X, Herzig CTA, Novak R, Bower W, Medley AM, Acosta AM, Knust B, Cantey PT, Pesik NT, Halsey ES, Cetron MS, Tong S, Marston BJ, Friedman CR. Coronavirus disease 2019 (COVID-19) in Americans aboard the Diamond Princess cruise ship. Clin Infect Dis. 2021; 72(10):e448-e457. doi: 10.1093/cid/ciaa1180.
16. Quigley, A., Nguyen, P., Stone, H., Lim, S., MacIntyre, C. Cruise ship travel and the spread of COVID-19 – Australia as a case study. International Journal of Travel Medicine and Global Health, 2020; 9(1): 10-18. doi: 10.34172/ijtmgh.2021.03
17. Sekizuka T, Kuramoto S, Nariai E, Taira M, Hachisu Y, Tokaji A, Shinohara M, Kishimoto T, Itokawa K, Kobayashi Y, Kadokura K, Kamiya H, Matsui T, Suzuki M, Kuroda M. SARS-CoV-2 genome analysis of Japanese travelers in Nile River cruise. Front Microbiol. 2020; 11:1316. doi: 10.3389/fmicb.2020.01316.
18. Sekizuka T, Itokawa K, Kageyama T, Saito S, Takayama I, Asanuma H, Nao N, Tanaka R, Hashino M, Takahashi T, Kamiya H, Yamagishi T, Kakimoto K, Suzuki M, Hasegawa H, Wakita T, Kuroda M. Haplotype networks of SARS-CoV-2 infections in the Diamond Princess cruise ship outbreak. Proc Natl Acad Sci U S A. 2020; 117(33):20198-20201. doi: 10.1073/pnas.2006824117.
19. Walker LJ, Codreanu TA, Armstrong PK, Goodwin S, Trewin A, Spencer E, Colquhoun SM, Stephens DM, Baird RW, Douglas NM, Cribb D, Owen R, Kelly P, Kirk MD. SARS-CoV-2 infections among Australian passengers on the Diamond Princess cruise ship: A retrospective cohort study. PLoS One. 2021;16(9):e0255401. doi: 10.1371/journal.pone.0255401.
20. Waltenburg MA, Pomeroy MA, Hughes L, Gold JAW, Mayer O, Vang A, Hallowell BD, Foster L, Xu K, Espinoza R, Hsieh K, Pieracci EG, Wuyke G, Da Silva J, McClung RP, Steinberg J, Westercamp M, Vallabhaneni S, Li J, Valderrama AL, Grimes GR, Harvey RR, Nett RJ, Stokes K, Lindstrom S, Miller AD, Griggs EP, Milucky JL, Bjork A, Albrecht V, Kuhnert WL, Gould CV, Knight NW, Nelson NP, Honein MA, Behravesh CB, CDC COVID-19 Investigation Team, Dubray CL, Marx GE. Testing for SARS-CoV-2 among cruise ship travelers repatriated to the United States, February–March 2020; medRxiv 2021.06.07.21258318; doi: https://doi.org/10.1101/2021.06.07.21258318
21. Yamagishi T, Kamiya H, Kakimoto K, Suzuki M, Wakita T. Descriptive study of COVID-19 outbreak among passengers and crew on Diamond Princess cruise ship, Yokohama Port, Japan, 20 January to 9 February 2020. Euro Surveill. 2020; 25(23):2000272. doi: 10.2807/1560-7917.ES.2020.25.23.2000272. Erratum in: Euro Surveill. 2020 Jun;25(24): PMID: 32553062; PMCID: PMC7403638.

22. Yamahata Y, Shibata A. Preparation for Quarantine on the Cruise Ship Diamond Princess in Japan due to COVID-19. JMIR Public Health Surveill. 2020; 6(2):e18821. doi: 10.2196/18821.
23. Yeh TY, Contreras GP. Viral transmission and evolution dynamics of SARS-CoV-2 in shipboard quarantine. Bull World Health Organ. 2021;99(7):486-495. doi: 10.2471/BLT.20.255752.

Environmental studies

1. Ahmed W, Bertsch PM, Angel N, Bibby K, Bivins A, Dierens L, Edson J, Ehret J, Gyawali P, Hamilton KA, Hosegood I, Hugenholtz P, Jiang G, Kitajima M, Sichani HT, Shi J, Shimko KM, Simpson SL, Smith WJM, Symonds EM, Thomas KV, Verhagen R, Zaugg J, Mueller JF. Detection of SARS-CoV-2 RNA in commercial passenger aircraft and cruise ship wastewater: a surveillance tool for assessing the presence of COVID-19 infected travellers. J Travel Med. 2020; 27(5):taaa116. doi: 10.1093/jtm/taaa116

2. Yamagishi T, Ohnishi M, Matsunaga N, Kakimoto K, Kamiya H, Okamoto K, Suzuki M, Gu Y, Sakaguchi M, Tajima T, Takaya S, Ohmagari N, Takeda M, Matsuyama S, Shirato K, Nao N, Hasegawa H, Kageyama T, Takayama I, Saito S, Wada K, Fujita R, Saito H, Okinaka K, Griffith M, Parry AE, Barnetson B, Leonard J, Wakita T. Environmental sampling for Severe Acute Respiratory Syndrome Coronavirus 2 during a COVID-19 outbreak on the Diamond Princess cruise ship. J Infect Dis. 2020; 222(7):1098-1102. doi: 10.1093/infdis/jiaa437. Erratum in: J Infect Dis. 2021; 223(3):540. PMID: 32691828; PMCID: PMC7454703.

Systematic reviews

1. Kordsmeyer AC, Mojtahedzadeh N, Heidrich J, Militzer K, von Münster T, Belz L, Jensen HJ, Bakir S, Henning E, Heuser J, Klein A, Sproessel N, Ekkernkamp A, Ehlers L, de Boer J, Kleine-Kampmann S, Dirksen-Fischer M, Plenge-Bönig A, Harth V, Oldenburg M. Systematic review on outbreaks of SARS-CoV-2 on cruise, navy and cargo ships. Int J Environ Res Public Health. 2021; 18(10):5195. doi: 10.3390/ijerph18105195.
