# Supplementary material for: Transmission of SARS-CoV-2 Associated with Cruise Ship Travel: A Systematic Review
Source: Trop Med Infect Dis. 2022 Oct 9;7(10):290. doi: 10.3390/tropicalmed7100290 (PMC9610645; doi:10.3390/tropicalmed7100290)
Supplement: Supplementary file 1 [file tropicalmed-07-00290-s001.zip › S6. USA citizens.pdf]

Supplementary Material S6

USA citizens repatriated from Diamond Princess

Waltenburg 2021	Plucinski 2020	Moriarty 2020
During February 16–17, 329 US citizens, residents, and their partners (subsequently referred to as repatriated travelers) voluntarily repatriated to the US from Japan. Of those repatriated, 328 were quarantined under federal authority at US military bases; one traveler was not quarantined based on documentation of having recovered from COVID-19 in Japan.	Of 3711 persons onboard, 437 were US citizens, residents, and non-American partners of citizens or residents	During February 16–23, nearly 1,000 persons were repatriated by air to their home countries, including 329 persons who returned to the US and entered quarantine or isolation. Overall, 111 (25.9%) of 428 U.S. citizens and legal residents did not join repatriation flights either because they had been hospitalized in Japan or for other reasons (428-111 = 317 ?)
Repatriated between 16-17 Feb 2020	Repatriated between 16–17 February	Repatriated 16 Feb (Fig. 1)
Retrospective, longitudinal evaluation of repatriated travelers where the outcome of interest was a positive test for SARS-CoV-2.	Retrospective cross-sectional survey of American passengers and crew using a standardized form including questions about demographics; symptoms; comorbidities; smoking; participation in group events during 3–5 February, the period just before ship-based quarantine; shore excursions during the voyage; and close contacts during the ship-based quarantine.	Report<br>3711 persons on DP, with 416 USA passengers, 0 crew
Specimens were collected by oropharyngeal (OP) and/or nasopharyngeal (NP) swab and tested by RT-PCR for SARS-CoV-2 [4]. Available RT-PCR results were obtained from the Japanese Ministry of Health, Labour, and Welfare, the Centers for	Specimens were collected by nasopharyngeal (NP) swab, oropharyngeal (OP) swab, or both. Specimens were tested by RT-PCR for SARS-CoV-2. Results from RT-PCR testing were provided by the Japanese Ministry	N/R

Disease Control and Prevention (CDC), and state public health laboratories	of Health, Labour, and Welfare (MHLW) and by the Centers for Disease Control and Prevention (CDC)	
The authors reviewed demographic and symptom data collected at the time of the first positive SARS-CoV-2 test.	The authors performed a survey of all Americans on the 20 January to 3 February sailing of the DP. Patients who were severely ill at the time of the survey and unable to answer questions were excluded (n = 10). Participants were interviewed 28 February to 18 March, approximately 2–5 weeks after disembarkation from the ship.	N/A
Of 328 quarantined repatriated travelers, 45 (14%) tested positive for SARS-CoV-2;	Of 437 American DP passengers and crew, 114 (26%) tested positive for SARS-CoV-2.	As of March 13, among 428 U.S. passengers and crew, 107 (25.0%) had positive test results for COVID-19; 11 U.S. passengers remain hospitalized in Japan (median age = 75 years), including seven in serious condition (median age = 76 years).
No crew	Crew were excluded from the risk factor analysis because there were few American crew, and their exposures were different from passenger exposures. Also, crew was excluded from the analysis of AR for cabin exposure categories; they did not undergo cabin quarantine.<br>Number of crew = 12 /437 USA citizens; interviewed = 6 crew members	After disembarkation of all passengers, crew members either completed a 14-day ship-based quarantine, were repatriated to and managed in their home country, or completed a 14-day land-based quarantine in Japan.<br>Country of residence of crew members:<br>Philippines 531 (51%); India 132 (13%); Indonesia 78 (7%); Other countries or unknown 304 (29%)

- Among 45 travelers, 28 (62%) had a positive RT-PCR result in Japan before repatriation.<br>- 25 (56%) of 45 travelers were asymptomatic or presymptomatic at the time of first positive SARS-CoV-2 result.	98 individuals had a positive RT-PCR result in Japan before repatriation.<br><br>- 21% of their infections were asymptomatic (Interviewed N = 229)	N/A
10 (22%) had negative results in Japan but then had a positive result in the US	10 who had negative results in Japan but then had a positive result in the US	N/A
7 (16%) who were never tested in Japan had a positive result in the US.	6 who were never tested in Japan and had a positive result in the United States.	N/A
SARS-CoV-2 negative test result N=283		N/A
N/A	Available samples from repatriated Americans testing positive in the US (n = 28) underwent whole genome sequencing for SARS-CoV-2	N/A
