# Supplementary material for: Transmission of SARS-CoV-2 Associated with Cruise Ship Travel: A Systematic Review
Source: Trop Med Infect Dis. 2022 Oct 9;7(10):290. doi: 10.3390/tropicalmed7100290 (PMC9610645; doi:10.3390/tropicalmed7100290)
Supplement: Supplementary file 1 [file tropicalmed-07-00290-s001.zip › S7. Timeline DP.pdf]

Supplementary Material S7

Timeline of events on Diamond Princess cruise ship

Date	Event	Notes
17 January 2020	• Hong-Kong case arrives to Japan	Yamagishi 2020
19 January 2020	• Index case developed symptoms; he disembarked on D5, and tested RT-PCR positive on 1 Feb 2020 (13 days later)<br>• Index case (Hong Kong case) – onset of cough	NIID 2020<br><br>Yamagishi 2020
20 January 2020 – D0	• The ship left Yokohama Port<br>• The 80-year-old Chinese gentleman, a resident of Hong Kong,China, visited Shenzhen in China's Guangdong Province for a few hours on January 10 (Nakazawa 2020). He took a flight from Hong Kong to Tokyo with his two daughters on 17 January 2020 and they embarked on the cruise ship in Yokohama, Tokyo on 20 January 2020 (Leung 2020)	https://wars.vote4.hk/en/cases/
21 January – D1	• N/R	
22 January – D2	• Passenger developed symptoms<br>• Kagoshima tour + bus<br>• The index case participated in a tour of Kagoshima, Japan during a shore-based stop (Yamagishi 2020)<br>• Other than the Hong Kong-related index case, symptom onset for the earliest confirmed case was 22 January	Kakimoto 2020<br>Plucinski 2020<br>Yamagishi 2020<br><br>Yamagishi 2020
23 January – D3	• Index case developed fever?<br>• Index case developed cough?	Kakimoto 2020<br>Sekizuka 2020
24 January – D4	• N/R	
25 January – D5	• Hong Kong tour + bus<br>• Index case disembarked in Hong Kong<br>• The ship visited Hong Kong	Plucinski 2020<br>NIIDS 2020<br>Yamagishi 2020
26 January – D6	• N/R	
27 January – D7	• Chan May Port, Vietnam tour + bus	Nakazawa 2020, Yamagishi 2020; Plucinski 2020
28 January – D8	• Cai Lan, Vietnam	Nakazawa 2020
29 January – D9	• 1 patient with symptoms	Kato 2020

	• Index case – fever	Hoshino 2020
30 January – D10	• Index case – developed fever<br>• Index case - attended a local hospital in Hong Kong	Yamahata 2020
31 January – D11	• Keelung, Taiwan	Yamagishi 2020
1 February – D12	• Naha, Japan; Okinawa port<br>• The Diamond Princess stops at Okinawa. A quarantine of the Diamond Princess (based on Quarantine Law) was ordered at Okinawa Port<br>• Index case – RT-PCR positive for SARS-COV-2<br>• Symptom onset date of the first crew member case was 1 February	Nakazawa 2020; Yamagishi 2020<br>NIID 2020; Sekizuka 2020<br>Yamagishi 2020
2 Feb – D13	• First crew member with symptoms (food service worker (Kakimoto 2020)<br>• 2 patients developed symptoms	Kakimoto 2020<br>Kato 2020
3 Feb – D14	• The ship arrived at Yokohama<br>• On board: 2666 passengers, 1045 crew<br>• Quarantine of the ship<br>• Health authorities in Japan reviewed logs at the onboard clinic for symptomatic (febrile or respiratory) patients and obtained respiratory specimens from them<br>• Starting on 3 Feb, quarantine officers took oropharyngeal samples from suspected cases and close contacts<br>• All symptomatic passengers were tested upon arrival in Yokohama (10 passengers), and those with positive results were disembarked February 4 and 5<br>• 3 crew members reported fever<br>• Group activities – music show, other shows	NIID 2020<br>NIID 2020<br>Nakazawa 2020<br>NIID 2020<br>Yamagishi 2020<br>Kakimoto 2020<br>Kakimoto 2020<br>Plucinski 2020
4 Feb – D15	• First crew member with positive RT-PCR<br>• Group activities – music show, other shows	Kakimoto 2020<br>Plucinski 2020
5 Feb – D16	• 35/127 passengers with known onset, developed symptoms before D16<br>• First 10/31 RT-PCR positive<br>• Passengers quarantined in their cabins for 14 days; the crew continued to maintain ship functions and support passengers for their food, clothing, and shelter-related needs<br>• Screening for the virus is implemented in the aged or people with chronic disease, even if asymptomatic	NIID 2020<br>Plucinski 2020, Yamahata 2020<br>NIID 2020<br>Nakazawa 2020

	• SARS-CoV-2 was detected through RT-PCR in 1 of the specimens taken on D14	NIID 2020
6 Feb – D17	• 10/71 RT-PCR positive<br>• First full day of quarantine<br>• Total number of positive tests 37/156 (4-6 February): 36 passengers, 1 crew<br>• Total number of positive tests: 20/102	Yamahata 2020<br>NIID 2020<br>Yamagishi 2020<br><br>Yamahata 2020
7 Feb – D18	• 41/171 RT-PCR positive<br>• 6/52 RT-PCR positive (1 passenger, 5 crew)<br>• Total number of positive tests: 61/273 (Yamahata 2020)<br>• Passengers and crew (although not all crew) were provided thermometers and asked to check their temperature several times daily<br>• A fever call center was established; persons on board were asked to call the center if they had a temperature >37.5°C<br>• The testing strategy changed; only suspected cases were tested (i.e., with symptoms), which had been increasing in number.<br>• The number of symptomatic passengers peaked (for cases with an available onset date, in 127 passengers and 51 crew	Yamahata 2020<br>Yamagishi 2020<br>Yamahata 2020<br>NIID 2020<br><br><br><br><br><br><br>NIID 2020
8 Feb – D19	• 3/6 RT-PCR positive<br>• 57/85 RT-PCR tested positive (52 passengers, 5 crew)<br>• Total number of positive tests: 64/279	Yamahata 2020<br>Yamagishi 2020<br>Yamahata 2020
9 Feb – D20	• 6/57 RT-PCR positive<br>• 44/65 RT-PCR tested positive (35 passengers, 9 crew)<br>• Total number of RT-PCR positive: 172/490 (152 passengers and 20 crew)<br>• Total number of positive tests: 70/337<br>• By February 9, 20 cases had occurred among the ship's crew members	Yamahata 2020<br>Yamagishi 2020<br>Yamagishi 2020<br>Yamahata 2020<br>Kakimoto 2020
10 Feb – D21	• 65/103 RT-PCR positive (Yamahata 2020)<br>• Total no of positive tests: 135/439 (Yamahata 2020)	Yamahata 2020<br>Yamahata 2020
11 Feb – D22	• Unavailable number of RT-PCR positive (Yamahata 2020)<br>• Start of RT-PCR testing for asymptomatic individuals<br>• Efforts shifted toward testing all passengers by PCR for SARS-CoV-2. Due to the number of persons on board, these efforts began with passengers over 80, then over 75, then over 70, etc.	Yamahata 2020<br>Plucinski 2020; Hoshino 2020<br>NIID 2020

	• First peak of symptomatic cases among crew peaked (for cases with an available onset date, in 127 passengers and 51 crew	NIID 2020
12 Feb – D23	• 39/53 RT-PCR positive<br>• Total number of positive tests: 174/492	Yamahata 2020<br>Yamahata 2020
13 Feb – D24	• A policy allows the aged to disembark, specifically, older people over the age of 80 years who are in cabins without windows and suffer from chronic illness<br>• Total number of positive tests: 218/713<br>• 44/221 RT-PCR positive<br>• Second peak of symptomatic cases among crew (for cases with an available onset date, in 127 passengers and 51 crew	Nakazawa 2020<br><br>Yamahata 2020<br>Yamahata 2020<br>NIID 2020
14 Feb – D25	• 67/217 RT-PCR positive<br>• Total number of positive tests: 285/930	Yamahata 2020<br>Yamahata 2020
15 Feb – D26	• Total number of positive tests: 285/930<br>• 15-17 Feb 2020 – 169/793 RT-PCR positive tests<br>• 15-17 Feb 2020 - pharyngeal specimens of 896 persons (880 passengers, 15 crew members, and 1 quarantine officer). PCR (RT-qPCR) test was positive for 148 individuals (138 passengers, 9 crew members, and 1 quarantine officer), indicating that the positivity rate was 16.4% (147 positive individuals [excluding the officer] of 895 subjects).	Yamahata 2020<br>Yamahata 2020<br>Sekizuka 2020
16 Feb – D27	• 70/289 RT-PCR positive<br>• Total number of positive tests: 335/1219<br>• 16 -23 February – 329 persons were repatriated by air to their home country (USA); they entered quarantine or isolation. Quarantine was used for persons who were exposed; isolation was used for persons who had positive test results for SARS-CoV-2<br>• 16–17 February, most Americans were repatriated voluntarily to the United States on chartered flights and quarantined under federal authority on US military bases	Yamahata 2020<br>Yamahata 2020<br>Moriarty 2020<br><br>Plucinski 2020
17 Feb – D28	• 99/504 RT-PCR positive<br>• Total number of positive tests: 454/1723<br>• Evacuation of Australian passengers	Yamahata 2020<br>Yamahata 2020<br>Quigley 2020
18 Feb – D29	• 88/681 RT-PCR positive	Yamahata 2020

	• Total number of positive tests: 542/2404	Yamahata 2020
19 Feb – D30	• End of quarantine period<br>• Disembarkation begins of passengers confirmed as not being infected<br>• Criteria for disembarkation of non-cases included 1) completion of a 14-day period without sharing a cabin with a confirmed case; 2) a negative result for a SARS-CoV-2 by PCR in the final days of the period; and 3) no relevant symptoms identified during a medical screening in the final day of the period.<br>• 79 RT-PCR positive<br>• 79/617 RT-PCR positive<br>• Total number of positive tests: 621/3011<br>• Repatriation of Australian citizens	NIID 2020<br>Nakazawa 2020<br>NIID 2020<br>NIID 2020<br>Yamahata 2020<br>Yamahata 2020
20 Feb – D31	• 13/52 RT-PCR positive<br>• Total number of positive tests: 634/3063<br>• Hong Kong citizens were air-evacuated from the Diamond Princess cruise ship on February 20 and February 21, 2020	Yamahata 2020<br>Yamahata 2002<br>Hung 2020
21 Feb – D32	• Total no of positive tests: 634/3063<br>• Infection had also been confirmed in 5 quarantined officers and health care workers<br>• Disembarkation of 970 asymptomatic passengers with negative test results is completed	Yamahata 2020<br>Nakazawa 2020<br>Nakazawa 2020
22 Feb – D33	• Approximately 1300 people, including crew members, foreigners waiting for charter aircraft, and passengers who were in close contact with or in the same room as a person who tested positive, are aboard<br>• Total number of positive tests: 634/3063	Nakazawa 2020<br>Yamahata 2020
23 Feb – D34	• 57/831 RT-PCR positive (Yamahata 2020)<br>• Total no of positive tests: 691/3894 (Yamahata 2020)	Yamahata 2020<br>Yamahata 2020
24 Feb – D35	• The remaining passengers disembark.	
25 Feb – D36	• N/R	
26 Feb – D37	• N/R	
27 Feb – D38	• N/R	
28 Feb - D39	• N/R	
29 Feb – D40	• N/R	

1 March – D41	• Disembarkation of 3711 individuals completed. The remaining crew members disembark.	Moriarty 2020
8 March	• Total number of cases: 697<br>• Total number of cases: 696	Sekizuka 2020<br>NIID 2020

References:

Hung IF, Cheng VC, Li X, Tam AR, Hung DL, Chiu KH, Yip CC, Cai JP, Ho DT, Wong SC, Leung SS, Chu MY, Tang MO, Chen JH, Poon RW, Fung AY, Zhang RR, Yan EY, Chen LL, Choi CY, Leung KH, Chung TW, Lam SH, Lam TP, Chan JF, Chan KH, Wu TC, Ho PL, Chan JW, Lau CS, To KK, Yuen KY. SARS-CoV-2 shedding and seroconversion among passengers quarantined after disembarking a cruise ship: a case series. Lancet Infect Dis. 2020; 20(9):1051-1060. doi: 10.1016/S1473-3099(20)30364-9.

Kakimoto K, Kamiya H, Yamagishi T, Matsui T, Suzuki M, Wakita T. Initial investigation of transmission of COVID-19 among crew members during quarantine of a cruise ship - Yokohama, Japan, February 2020. MMWR Morb Mortal Wkly Rep. 2020 Mar 20;69(11):312-313. doi: 10.15585/mmwr.mm6911e2. Erratum in: MMWR Morb Mortal Wkly Rep. 2020; 69(13):389.

Kato H, Shimizu H, Shibue Y, Hosoda T, Iwabuchi K, Nagamine K, Saito H, Sawada R, Oishi T, Tsukiji J, Fujita H, Furuya R, Masuda M, Akasaka O, Ikeda Y, Sakamoto M, Sakai K, Uchiyama M, Watanabe H, Yamaguchi N, Higa R, Sasaki A, Tanaka K, Toyoda Y, Hamanaka S, Miyazawa N, Shimizu A, Fukase F, Iwai S, Komase Y, Kawasaki T, Nagata I, Nakayama Y, Takei T, Kimura K, Kunisaki R, Kudo M, Takeuchi I, Nakajima H. Clinical course of 2019 novel coronavirus disease (COVID-19) in individuals present during the outbreak on the Diamond Princess cruise ship. J Infect Chemother. 2020;26(8):865-869. doi: 10.1016/j.jiac.2020.05.005

Moriarty LF, Plucinski MM, Marston BJ, Kurbatova EV, Knust B, Murray EL, Pesik N, Rose D, Fitter D, Kobayashi M, Toda M, Cantey PT, Scheuer T, Halsey ES, Cohen NJ, Stockman L, Wadford DA, Medley AM, Green G, Regan JJ, Tardivel K, White S, Brown C, Morales C, Yen C, Wittry B, Freeland A, Naramore S, Novak RT, Daigle D, Weinberg M, Acosta A, Herzig C, Kapella BK, Jacobson KR, Lamba K, Ishizumi A, Sarisky J, Svendsen E, Blocher T, Wu C, Charles J, Wagner R, Stewart A, Mead PS, Kurylo E, Campbell S, Murray R, Weidle P, Cetron M, Friedman CR; CDC Cruise Ship Response Team; California Department of Public Health COVID-19 Team; Solano County COVID-19 Team. Public Health Responses to COVID-19 Outbreaks on Cruise Ships - Worldwide, February-March 2020. MMWR Morb Mortal Wkly Rep. 2020; 69(12):347-352. doi: 10.15585/mmwr.mm6912e3.

NIID - Expert Taskforce for the COVID-19 Cruise Ship Outbreak. Epidemiology of COVID-19 outbreak on cruise ship quarantined at Yokohama, Japan, February 2020. Emerg Infect Dis. 2020; 26(11):2591-2597. doi: 10.3201/eid2611.201165.

Nakazawa E, Ino H, Akabayashi A. Chronology of COVID-19 cases on the Diamond Princess cruise ship and ethical considerations: a report from Japan. Disaster Med Public Health Prep. 2020;14(4):506-513. doi:10.1017/dmp.2020.50

Plucinski MM, Wallace M, Uehara A, Kurbatova EV, Tobolowsky FA, Schneider ZD, Ishizumi A, Bozio CH, Kobayashi M, Toda M, Stewart A, Wagner RL, Moriarty LF, Murray R, Queen K, Tao Y, Paden C, Mauldin MR, Zhang J, Li Y, Elkins CA, Lu X, Herzig CTA, Novak R, Bower W, Medley AM, Acosta AM, Knust B, Cantey PT, Pesik NT, Halsey ES, Cetron MS, Tong S, Marston BJ, Friedman CR. Coronavirus Disease 2019 (COVID-19) in Americans aboard the Diamond Princess cruise ship. Clin Infect Dis. 2021; 72(10):e448-e457. doi: 10.1093/cid/ciaa1180.

Quigley, A., Nguyen, P., Stone, H., Lim, S., MacIntyre, C. Cruise ship travel and the spread of COVID-19 – Australia as a case study. International Journal of Travel Medicine and Global Health, 2020; 9(1): 10-18. doi: 10.34172/ijtmgh.2021.03

Sekizuka T, Itokawa K, Kageyama T, Saito S, Takayama I, Asanuma H, Nao N, Tanaka R, Hashino M, Takahashi T, Kamiya H, Yamagishi T, Kakimoto K, Suzuki M, Hasegawa H, Wakita T, Kuroda M. Haplotype networks of SARS-CoV-2 infections in the Diamond Princess cruise ship outbreak. Proc Natl Acad Sci U S A. 2020; 117(33):20198-20201. doi: 10.1073/pnas.2006824117.

Yamagishi T, Kamiya H, Kakimoto K, Suzuki M, Wakita T. Descriptive study of COVID-19 outbreak among passengers and crew on Diamond Princess cruise ship, Yokohama Port, Japan, 20 January to 9 February 2020. Euro Surveill. 2020; 25(23):2000272. doi: 10.2807/1560-7917.ES.2020.25.23.2000272. Erratum in: Euro Surveill. 2020 Jun;25(24): PMID: 32553062; PMCID: PMC7403638.

Yamahata Y, Shibata A. Preparation for quarantine on the cruise ship Diamond Princess in Japan due to COVID-19. JMIR Public Health Surveill. 2020; 6(2):e18821. doi: 10.2196/18821.
