# Supplementary material for: Transmission of SARS-CoV-2 Associated with Cruise Ship Travel: A Systematic Review
Source: Trop Med Infect Dis. 2022 Oct 9;7(10):290. doi: 10.3390/tropicalmed7100290 (PMC9610645; doi:10.3390/tropicalmed7100290)
Supplement: Supplementary file 1 [file tropicalmed-07-00290-s001.zip › S9. Genomic sequencing.pdf]

S9. Genomic sequencing studies.

**Study**	**Methods for genome sequencing**	**Phylogenetic analysis**	**Results**
Abe 2022	All positive samples that showed high viral titers (Ct<30 by RT-qPCR) were subjected to whole-genome sequencing; 94 complete genome sequences were obtained for the CA cluster. Multiplex PCR was performed using primer sets designed by the online program Primal Scheme, with an amplicon size of 450 bp. The template sequence was derived from the Wuhan-Hu-1 strain. Extracted viral RNA was reverse-transcribed using SuperScript IV Reverse Transcriptase (Thermo Fisher Scientific) combined with random hexamers. Reverse-transcribed complementary DNA was used to perform multiplex PCR with Q5 High-Fidelity DNA Polymerase (New England Biolabs, Ipswich, MA, USA). Libraries were prepared using 40–500 ng of multiplex PCR products and an NEBNext Ultra II DNA Library Prep kit (New England Biolabs) in combination with NEBNext Multiplex Oligos for Illumina	The Costa Atlantica strains showed three main clusters; the CA-A cluster was genetically closest to the reference strain, Wuhan-Hu-1, possibly indicating that the CA-A cluster was the haplotype originally introduced into the CA cluster. CA-A was not a large cluster and only two infected individuals possibly played a central role in spreading SARS-CoV-2 in the CA-A cluster. The core populations of clusters CA-B and CA-C included more than 10 individuals, indicating that these clusters were caused by superspreading event-like infections.	The authors report 1175 mutations (number/strain 12.5), 755 amino acid substitutions (number/strain 8.03). Comparison with other settings: DP - 71 samples, 149 mutations (number/strain 2.1), 110 amino acid substitutions (number/strain 1.55). Fiji Japan - 82 samples, 1191 mutations (number/strain 14.52), 612 amino acid substitutions (number/strain 7.46)<br>CA strains formed one large cluster without any other reference strains, indicating that CA strains originated from one ancestral strain introduced on the cruise ship and circulated in a highly isolated environment. However, the number of mutations in the DP cluster was remarkably lower than that in the CA cluster, indicating environmental differences between the two cruise ships. The SARS-CoV-2 circulation on the CA might have begun as asymptomatic cases, and more asymptomatic infection cases had been unrecognized in the CA cruise ship, generating much more genetic diversity compared with that in the DP cluster.

	(New England Biolabs). After a quantitative inspection of each library using an NEBNext Library Quant Kit for Illumina (New England Biolabs), the authors used the large-scale sequencing service GeneNex (Chemical-dojin, Tokyo, Japan) to obtain complete genome sequences of SARS-CoV-2. Mapping of the paired-end reads was performed on CLC GenomicsWorkbench 11.0.1 software (Qiagen) using a whole-genome sequence of the Wuhan-Hu-1 strain as a template. Consensus sequences were extracted and aligned with reference strains using BioEdit 7.0.5.3 software. Sanger sequencing was performed to obtain complete GS of several strains that showed short ambiguous sequences.		
Hoshino 2021	The authors retrieved from the GISAID database all publicly available SARS-CoV-2 genome sequences with clinical information as of 7 August 2020 (n = 78,448). In total, 74 SARS-CoV-2 sequences from infected persons on the DP were identified. Sequences with multiple ambiguous nucleotides (>1%) and long gaps in the coding region were excluded. The authors	The authors performed phylodynamic analyses to investigate the transmission dynamics of SARS-CoV-2 on DP.<br>The general time-reversible model with invariable sites and γ distribution among site rate variation (GTR+G+I) was selected as the bestfit model for the reference dataset using ModelTest NG.<br>Phylogenetic trees were constructed using RAxML (v.8.2.9) with 1000 replicates using the maximum likelihood (ML) algorithm.	The authors estimated that the outbreak originated on 21 January, which coincided with the date when the presumed index case from Hong Kong boarded the ship. The study reports that the effective population size began to increase around 30 January and exponentially surged from 2 February, before the start of quarantine on 5 February. After quarantine, the transmission of the virus continued slowly. Also, the authors found that, although branch

	retained and analyzed 67 sequences that were collected between 10 -17 February 2020.	Trees were created using FigTree (v.1.4.4) and were re-rooted using the earliest sample (IPBCAMS-WH-01, GenBank entry: MT019529.1). Bayesian phylogenetic analysis was conducted using a constant coalescent model and the strict molecular clock model implemented in BEAST (v.1.10.4).<br>The SARS-CoV-2 population dynamics on the DP were inferred using a Bayesian skyline plot coalescent model with the strict molecular clock model implemented in BEAST (v.1.10.4). The GTR+G+I substitution model was used, and the prior for the nucleotide substitution rate was set based on the values obtained in the rate estimation. The MCMC run contained 100 million states, with sampling every 10,000 states. The ESS values were >200 after a 10% burn-in; three independent runs were combined using LogCombiner (v. 1.10.4) implemented in the BEAST package. The effective population sizes were plotted using R (v.4.0.2, R Core Team, 2018)	bootstrap values were low, all sequences from the DP clustered with some isolates collected in other countries.
Murata 2021	SARS-CoV-2 genomic RNA was extracted from culture supernatants and selected nasopharyngeal samples with Cp values less than 34, using a QIAamp Viral RNA minikit (Qiagen). The viral RNA extracted from culture supernatants was subjected to direct MiSeq sequencing. A 200-bp fragment library ligated with bar-coded adapters was constructed using an NEBNext Ultra RNA library prep kit for Illumina v1.2 (New England	Sequences extracted from each genome were aligned to the reference genome and inspected.<br>Variants with allele frequencies of ≥70% were identified as SNVs. Variant detection was performed using VarScan (20) in addition to manual inspection. An intrahost single nucleotide variation (iSNV) was defined as a variation with an allele frequency of .10% and sequencing depth of ≥1,000. A web application tool (http://giorgilab.dyndns.org/coronapp/) was also used for variant detection and annotation of mutated genes.	In the study by Murata et al. [Murata], the authors extracted SARS-CoV- 2 genomic RNA from culture supernatants and selected nasopharyngeal samples with Cp values < 34. The complete GS of 4 sequential specimens collected from one carrier (Carrier_1), as well as those of 9 specimens obtained from his cabinmate, and six others, were obtained and analyzed. All SARS-CoV-2 strains belonged to clade 19A; they presented a single nucleotide mutation at position 11083 (G11083T transversion),

	Biolabs) according to the manufacturer's instructions. The cDNA library was purified using Agencourt AMPure XP magnetic beads (Beckman Coulter).<br>Nucleotide sequencing was performed on an Illumina MiSeq sequencer (Illumina) using a MiSeq reagent kit v2 (Illumina) to produce 151 paired-end reads. Data analysis of the direct MiSeq sequencing was carried out using CLC Genomics Workbench v8.0.1 (CLC Bio). Contigs were assembled from the obtained sequence reads (trimmed) by de novo assembly.<br>To further refine the contigs, the sequence reads were mapped back to the assembled contigs. In order to obtain longitudinal sequence data, SARS-CoV-2 genomes were amplified from selected nasopharyngeal specimens with Cp values less than 34 from which viable viruses were not isolated according to the PCR protocol of the ARTIC network or using a QIAseq SARS-CoV-2 primer panel (Qiagen). Sequencing libraries of the amplicons were prepared using a QIAseq FX DNA library kit (Qiagen), and the libraries were analyzed with an Illumina MiSeq sequencer, using the MiSeq reagent kit V2, for		leading to a nonsynonymous amino acid substitution (Leu37Phe) in nonstructural protein 6 (nsp6), as previously described by Sekizuka et al. [Sekizuka 2020].<br>The GS analysis of SARS-CoV-2 samples consecutively obtained from Carrier_1, who shed viable virus for 15 days, identified in the sample collected on day 15, the emergence of two novel single nucleotide variants (C8626T transition and C18452T transition), with the latter corresponding to an amino acid substitution in nonstructural protein 14. The former mutation in the nsp4 gene was synonymous, whereas the latter led to a nonsynonymous amino acid substitution (Ala138Val) in nsp14, which possesses a 39-to-59 exonuclease activity. None of these mutations were found in samples collected from the cabinmate of Carrier_1 and others.

	300 cycles. Amplicon sequences were mapped to the reference sequence (MN908947.3), and consensus sequences were obtained according to the Utah Department of Health ARTIC/Illumina Bioinformatic Workflow with minor modifications to include reads without primers. Consensus sequences obtained from the workflow were confirmed by reading the mapping files (bams) using Integrative Genomics Viewer (IGV).		
Plucinski 2020	Available RT-PCR samples from repatriated Americans testing positive in the United States (n = 28) underwent whole genome sequencing for SARS-CoV-2.	A phylogenetic tree was built using the global database of SARS-CoV-2 sequences available before 20 March 2020. A median joining network of observed haplotypes was generated using the subset of sequences from samples collected from those on the DP including 69 previously reported sequences from Japan and Hong Kong and the 28 sequences from this investigation.	All SARS-CoV-2 genome sequences clustered in the B group of the global phylogenetic tree, a group dominated by early sequences from China and containing all previously reported genome sequences from the DP.<br>All genome sequences presented the same G11083T mutation reported in the presumed Hong Kong index case, consistent with a single index case leading to this cruise ship outbreak.<br>Among the 28 sequences, 12 were from 6 pairs of individuals who had close contact. In all instances, pairs of linked genomes (Links A-F) grouped closely to one another within the haplotype network. Linked sequences were separated by only 0–2 single nucleotide variants (SNVs) compared with 0–9 SNVs among all sequences from DP. Two pairs (Link E and Link C) had identical

			sequences, whereas 4 pairs (Links A, B, D, and F) had unique sequences with 1–2 SNVs separating the pairs.
Sekizuka 2020	All 148 RT-qPCR positive RNA samples were subjected to the PrimalSeq protocol to generate enriched cDNA of the SARS-CoV-2 genome, followed by next-generation sequencing (NGS).<br>In total, 70 whole-genome sequences have been determined. The Cq limit for successful genome sequence determination was around 32 (corresponding to a virus copy number of less than 300).<br>Authors used a multiplexed PCR primer set, which was proposed by the Wellcome Trust ARTIC Network. The PCR products from the same clinical samples were pooled, purified, and subjected to Illumina library construction using the QIAseq FX DNA Library Kit (Qiagen).<br>The NextSeq 500 platform (Illumina) was used for sequencing the indexed libraries.	The 73 genome sequences (including 3 sequences of DP isolates deposited in the GISAID were compared with the Wuhan-Hu-1 (isolated on December 26, 2019 in China) genome sequence as a reference.<br>Maximum-likelihood (ML) phylogenetic analysis including other SARS-CoV-2 genome sequences that are publicly available on GISAID and haplotype networks from genomic SNVs (HN-GSNVs) were used to map the genotypes of the SARS-CoV-2 isolates that disseminated in the DP cruise ship after isolation of the passengers on February 5, 2020<br>To decode the genealogies of the whole genome of SARS-CoV-2, authors generated HN-GSNVs to highlight the clonality of DP isolates.<br>Gap-containing sequences in the core region were excluded; sequences of 243 isolates from the GISAID database were used in subsequent analyses. An ML phylogenetic analysis with SNVs was performed using iqtree v1.6.12 (18) with an ultrafast	In total, 70 whole-genome sequences were determined (9/22 RT-PCR positive symptomatic cases; 65/125 RT-PCR positive asymptomatic cases; 1 RT-PCR positive case with symptoms not reported).<br>The frequencies of SNVs suggested that all 73 isolates (70 from DP and 3 from GISAID) shared an SNV; the G nucleotide at the 11,083 position on the Wuhan-Hu-1 genome sequence was mutated to T (G11083T transversion), leading to a nonsynonymous aminoacid substitution (Leu37Phe) in the nsp6 protein. Some additional minor SNVs were identified throughout the genome sequence.<br>The authors identified several nucleotide variations: 52 SNVs among DP isolates (n = 73) and 449 SNVs among all tested isolates, including entries from GISAID (n = 412). Pyrimidine transition, particularly cytosine (C) to thymine (T), made up a large portion of the SNVs. There

	Then, the authors used data from another round of 3-kb RT-PCR assays covering the whole genome of SARS-CoV-2 (29.9 kb) with 10 PCR amplicons. The 3-kb RT-PCR multiplexed amplicons were mixed in a single tube and subjected to shotgun NGS on the NextSeq 500 platform (Illumina) as described for the ARTIC library.<br>Thirty nucleotides corresponding to the multiplex primer sequences at both ends of NGS reads were trimmed for further analysis. The obtained NGS reads were mapped to the SARS-CoV-2 Wuhan-Hu-1 reference genome sequence (29.9 kb single-strand RNA; GenBank ID: MN908947), resulting in a specimen-specific SARS-CoV-2 genome sequence fully mapped with a read depth of at least ≥100× from the 99- to 29,796-nt region of Wuhan-Hu-1 genome sequence. The full genome sequence was determined by de novo assembly using A5-miseq v.20140604.	bootstrap test parameter, followed by visualization using iTOL v4 (19). The genome sequences of the DP isolates were aligned using MAFFT with sequences retrieved from the database, followed by extraction of SNV and deletion sites. The SNV median-joining network analysis was performed using the PopART software.	was a relatively low frequency of transversion mutations, but the guanine (G) to thymine (T) transversion, in particular, was observed in both DP isolates and other SARS-CoV-2 isolates. The number of nonsynonymous mutations (62%) was higher than that of synonymous mutations (33%) and mutations in noncoding/intergenic regions (5%).<br>The finding that all isolates in the DP cluster exhibit G11083T transversion strongly suggest that SARS-CoV-2 dissemination in the DP could have originated from a single introduction event before the quarantine started on February 3.<br>Focusing on the HN-GSNVs map on only the DP isolates revealed that the 29 isolates of the DP-A cluster was predominant, indicating that the DP-A cluster was the ancestral haplotype for subsequent transmission. Although further explosive spreading might have been prevented after the quarantine, some of the subsequent progeny clusters, including DP-B (five isolates) and DP-C (six isolates), may have formed via transmission through hidden links, such as mass-gathering events in the recreational areas and direct transmission among passengers who shared cabins. Additionally, 33 patients (45%) not included in the DP-A, -B, or -C clusters had

			unique SARS-CoV-2 haplotypes with patient-specific unique SNVs and/or deletions.<br>The authors deposited all whole genome sequences (fasta) data to GISAID [Sekizuka a].
Sekizuka b 2020	Whole genome sequences of SARS-CoV-2 was obtained by PrimalSeq protocol to enrich cDNA of SARS-CoV-2 genome by multiplex RT-PCR amplicons using a multiplexed PCR primer set which was proposed by Wellcome Trust ARTIC Network.<br>The PCR products from same clinical sample was pooled, purified and subjected for Illumina library construction using QIAseq FX DNA Library Kit. NextSeq 500 platform (Illumina, San Diego, USA) was used for sequencing the indexed libraries.<br>The NGS reads were mapped to the Wuhan-Hu-1 reference genome sequence (29.9 kb ss-RNA; GenBank ID: MN908947), resulting to the specimen-specific SARS-CoV-2 genome sequence by fully mapping on the reference. The mapped reads of SARS-CoV-2 sequences were	The nearly full-length genome sequence (≥ 29 kb) of SARSCoV-2 were retrieved from GISAID EpiCoV database in March 10, 2020, followed by multiple alignment using MAFFT v7.222. The poorly aligned regions in 5′ and 3′ end was trimmed; authors determined that the core regions were from 99 to 29,796 nt position against Wuhan-Hu-1 genome sequences.<br>Gap-containing sequences in the core region were excluded; sequences of 1,507 isolates in GISAID database were used in subsequent analyses (updated on March 30, 2020).<br>The genome sequences were aligned using MAFFT program together with sequences retrieved from database, followed by extraction of SNV and deletion sites.<br>The SNV median-joining network analysis was performed by PopART software3.	Authors reported data on GS from five passengers from two Nile River cruises. They evaluated viral GS from SARS-CoV-2-positive travelers who returned to Japan from Egypt. Three passengers who traveled aboard the same ship presented identical SARS-CoV-2 genome sequences. The isolate showed a close lineage with European isolates, with several SNVs.<br>In addition, the authors report a couple of passengers that boarded a different cruise ship but had the same Tokyo to Cairo flight as one of the travelers reported above. These two SARS-CoV-2 isolates showed identical GS but distinct from the genome sequences of the first three travelers by only one. The authors compared the GS of the passengers with the only two genome sequences of SARS-CoV-2 isolates in Egypt available in GISAID at the time of the study. The haplotype network exhibited that one of the passengers of the first cruise and the two passengers from

	assembled using A5-miseq v.20140604 to determine the full genome sequence.<br>The SNV sites and marked heterogeneity were extracted by the read-mapping at ≥10 × depth and from 99 to 29,796 nt region ofWuhan-Hu-1 genome sequence.		the second cruise was closely related to Egypt isolates with 2 or 3 SNVs.
Yeh 2021	The authors retrieved from the GISAID database 28 SARS-CoV-2 genome sequences and annotations of the isolates from the cruise ship, as well as the reference genomes of SARS-CoV-2 isolates PBCAMS-WH-04 (accession no. MT019532), WIV04 (MN996528), Hu-1 (NC045512) and WHU01 (MN98868). The samples were collected between 10 and 25 February 2020.	The authors analysed evolution dynamics of SARS-CoV-2 using computational tools of phylogenetics, natural selection pressure and genetic linkage. They aligned FASTA files of viral sequences using MAFFT 7 software (Kazutaka Katoh, Research Institute for Microbial Diseases, Osaka, Japan). To analyse phylogenetic relationships between viral sequences, they used the neighbour-joining method and Jukes–Cantor substitution model with setting bootstrap resampling number as five. The rectangular phylogenetic tree was generated using Archaeopteryx with Java plug-in of MAFFT 7. For radial phylogenetic tree, they first exported the tree file as Newick format by MAFFT; the FigTree software (version 1.4.2, Andrew Rambaut, University of Edinburgh, Edinburgh, United Kingdom of Great Britain and Northern Ireland) was used to transform and display the cladogram. To illustrate the viral subgroups with 11803G > T mutation, the authors rooted an unrooted tree by introducing the bat SARS-like CoV WIV16 (accession no. KT444582) as an outgroup virus.	The authors identified a total of 24 new viral mutations across 64.2% (18/28) of samples; the virus evolved into at least five subgroups. Increased positive selection of SARS-CoV-2 were statistically significant during the quarantine (Tajima's D: −2.03, $P < 0.01$; Fu and Li's D: −2.66, $P < 0.01$; and Zeng's E: −2.37, $P < 0.01$). Linkage disequilibrium analysis confirmed that ribonucleic acid (RNA) recombination with the11083G > T mutation also contributed to the increase of mutations among the viral progeny. The SARS-CoV-2 outbreak in the cruise most likely originated from either a single person infected with a virus variant identical to the WIV04 isolates, or simultaneously with another primary case infected with a virus containing the 11083G > T mutation. The findings indicate that the 11083G > T mutation of SARS-CoV-2 spread during shipboard quarantine and arose through de novo RNA recombination under positive selection pressure.

		For selection pressure, the authors used MEGA7 software to calculate Tajima's D test of neutrality.	

Abbreviations: RT-PCR - real time reverse transcription–polymerase chain reaction; SNV - single nucleotide variants
